# Taxonomic Composition of *Iris* Subser. *Chrysographes* (Iridaceae) Inferred from Chloroplast DNA and Morphological Analyses

**DOI:** 10.3390/plants10112232

**Published:** 2021-10-20

**Authors:** Eugeny V. Boltenkov, Elena V. Artyukova, Anna Trias-Blasi

**Affiliations:** 1 Botanical Garden-Institute, Far Eastern Branch, Russian Academy of Sciences, 690024 Vladivostok, Russia; 2 Federal Scientific Center of the East Asia Terrestrial Biodiversity, Far Eastern Branch, Russian Academy of Sciences, 690022 Vladivostok, Russia; artyukova@biosoil.ru; 3 Royal Botanic Gardens, Kew, Richmond TW9 3AE, UK; A.TriasBlasi@kew.org

**Keywords:** chloroplast DNA, *Chrysographes*, *Iris*, molecular phylogeny, morphometry, taxonomy

## Abstract

The species of *Iris* subser. *Chrysographes* are herbaceous perennials found mainly in southwestern and central China and also in the Eastern Himalayas. To date, six species have been recognized in this group. In the framework of its taxonomic revision, we have carried out molecular and morphological studies. For this, we have sequenced four chloroplast DNA regions (*trnS*–*trnG*, *trnL–trnF*, *rps4–trnS*^GGA^, and *psbA*–*trnH*) for 25 samples across the major distribution ranges of the six species. Our phylogenetic analyses evidence that *I.* subser. *Chrysographes* is indeed a monophyletic group, which is sister to *I.* subser. *Sibiricae*. Within *I.* subser. *Chrysographes*, we have recovered four divergent lineages further supported by diagnosable morphological traits and geographical distributions. In this context, our data confirm the recognition of *I. clarkei*, *I. delavayi*, and *I. wilsonii* in their traditional concepts. Furthermore, both molecular and morphological data support the close affinities and similar distribution ranges of *I. bulleyana*, *I. chrysographes*, and *I. forrestii*, which suggests including *I. chrysographes* and *I. forrestii* as color forms in *I. bulleyana*. A revised taxonomic treatment for the group, including the notes on the species distributions and habitats, and also an identification key to the species are provided.

## 1. Introduction

While revising the series *Sibiricae* (Diels) G.H.M.Lawr. of the section *Limniris* Tausch of the genus *Iris* L., we found that the taxonomy of this group remains unclear [1]. *Iris* ser. *Sibiricae* includes rhizomatous herbaceous perennials morphologically distinguished mainly by their hollow flowering stems (except for *I. clarkei* Baker ex Hook.f.). The infrageneric taxon *Sibiricae* was proposed by Diels [2] as a subsection including eight species. Simonet [3] subdivided this subsection into two groups on the basis of their chromosome numbers. Nevertheless, the distinctness of the two subseries, recognized on morphological ground and from their distributions [4,5], gained support from molecular studies [6,7].

**Figure 1:**
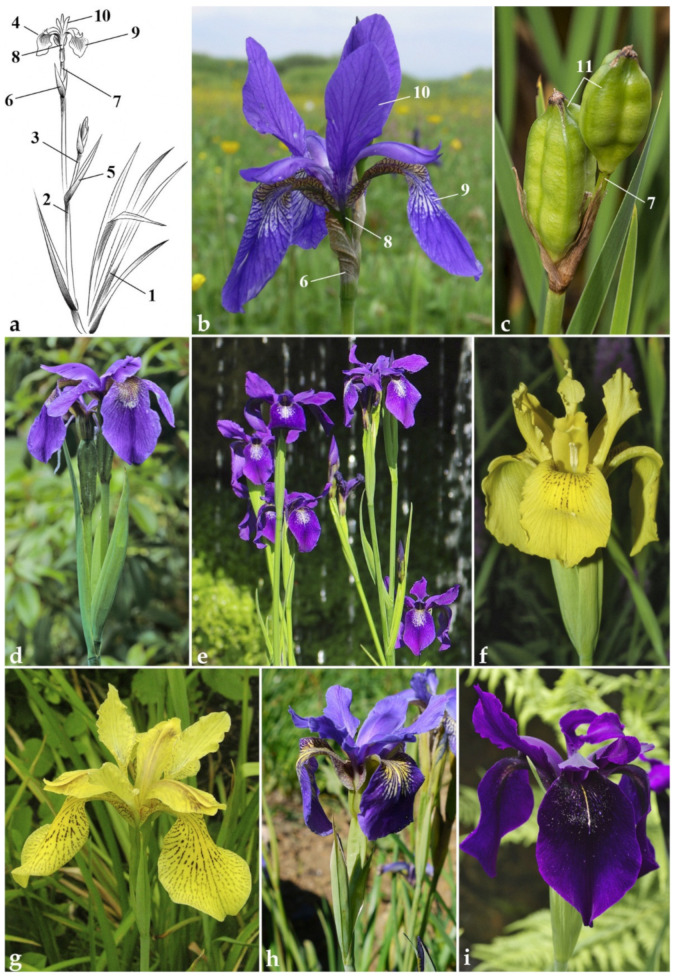
Morphological diversity in *Iris* ser. *Sibiricae*: (**a**) habitus; (**b**) *I. sibirica*, a flower (Carpathian Biosphere Reserve, Ukraine; by O. Kolesnyk); (**c**) *I. sibirica*, fruit (Russia, Karachay-Cherkessia; by T. Gaidash); (**d**) *I. clarkei*, flowers (cultivated plant; by RBGE staff); (**e**) *I. delavayi*, flowering stems (cultivated plant; by R. Wilford); (**f**) *I. wilsonii*, a flower (cultivated plant; by O. Fragman-Sapir); (**g**) *I. forrestii*, a flower (cultivated plant; by N. Shevyreva); (**h**) *I. bulleyana*, a flower (Litiping Plateau, Weixi Lisu Autonomous County, Yunnan Province, China; by I. Illarionova); (**i**) *I. chrysographes*, a flower (cultivated plant; by R. Wilford). Marks: 1, rosette leaf; 2, flowering stem; 3, branch; 4, terminal flower; 5, upper cauline leaf; 6, outer bract; 7, pedicel; 8, tube; 9, fall; 10, standard; 11, fruits.

*Iris sibirica* L., *I. sanguinea* Hornem., and *I*. *typhifolia* Kitag., with their 2n = 28 chromosomes [3,8,9], have been recognized in the autonymic subseries of *I*. ser. *Sibiricae* [10,11] known to horticulturists under the common name “Siberian irises” [12]. In a recent study [1], we confirmed that *I.* subser. *Sibiricae* includes only the highly variable *I. sibirica* (Figure 1b,c). It is the most widespread Eurasian species of Iridaceae, occurring from central and eastern Europe to the Russian Far East.

*Iris* subser. *Chrysographes* (Simonet) L.W.Lenz species are distributed from northeastern India and southwestern China, Nepal, and Bhutan to northern Myanmar, and are found at mid- to high elevations [11,13,14]. The name *Chrysographes* was proposed by Simonet [3] as a subsection for the species with the chromosome number 2n = 40. Alternatively, *Chrysographes* was referred to the genus *Limniris* (Tausch) Rchb. as a series [15], or a section [16], and is commonly known as “Sino-Siberians” among horticulturists [12]. Morphologically (see Figure 1), the *I.* subser. *Chrysographes* species is characterized by having longer bracts and perianth tube, and flowers without the venation characteristic of *I. sibirica* [5,11,17].

As is recognized, *I.* subser. *Chrysographes* comprises eight species, *I. bulleyana* Dykes, *I. chrysographes* Dykes, *I. clarkei*, *I. delavayi* Micheli, *I. dykesii* Stapf, *I. forrestii* Dykes, *I. phragmitetorum* Hand.-Mazz., and *I. wilsonii* C.H.Wright; however, the taxonomy of some of them was considered controversial [4,10,11,13,14,18]. As a result of a preliminary taxonomic study based on an analysis of the original material [19], six species were attributed to *I.* subser. *Chrysographes* (Figure 1d–i). Among them, *I. delavayi*, *I. wilsonii*, *I. bulleyana*, and *I. forrestii* were described from cultivated plants. The examination of the original material has shown that *I. delavayi*, as well as *I. bulleyana* and *I. forrestii* (both from Lijiang), originated from the northwestern Yunnan Province, China, while *I. wilsonii* originated from the northwestern Hubei Province, China [19]. *Iris clarkei* and *I. chrysographes* were described from plants collected in the wild, from eastern India and the Sichuan Province (China), respectively.

Nevertheless, it was indicated that there remains a great deal of confusion about the *I.* subser. *Chrysographes* species from southwestern China, and a thorough revision is required to resolve the taxonomy of this group [10,20,21]. A doubt was expressed about whether *I. bulleyana* is an independent species. For years, *I. bulleyana* was suggested to be a natural hybrid between *I. forrestii* and *I. chrysographes* since, in the wild, these species grow in the same regions, and their natural hybridization is possible [4,22]. However, fieldwork in the Yunnan Province (China) showed that *I. bulleyana* is a true species and is sometimes found in associations with *I. chrysographes* or *I. forrestii* [11,14]. According to another point of view [21], *I. chrysographes* and *I. bulleyana* are considered extreme forms of a single species. In particular, Noltie [10] (p. 300) noted that no differentiation can be made between the two yellow-flowered *I. forrestii* and *I. wilsonii* when based solely on herbarium material, *I. bulleyana* is merely a purple-flowered form of the same species, and that *I. chrysographes* and *I. delavayi* are probably distinct species, though it is very difficult to distinguish between them in a herbarium.

In recent decades, much research has been conducted on plant barcoding using DNA sequences, including those of *Iris* [23,24,25,26,27,28,29]. Chloroplast DNA has been extensively used to investigate phylogenetic relationships in plants [30]. Sequences of many cpDNA noncoding regions, including introns and intergenic spacers, have been used to assess interspecific relationships and suggested as molecular markers for species identification [31]. The regions *trnS*–*trnG*, *trnL*–*trnF*, *rps4*–*trnS*^GGA^, and *psbA*–*trnH* of cpDNA have proven to be useful as phylogenetic markers in the genus *Iris* [1,32,33,34].

Accordingly, in the framework of taxonomic studies carried out on *I*. ser. *Sibiricae* [1,19,35], the aims of the present study are as follows: (1) to resolve the phylogenetic relationships of the *I*. ser. *Sibiricae* species and some other series of *I*. sect. *Limniris* using four cpDNA regions; (2) to determine a possible number of putative chloroplast lineages within *I*. subser. *Chrysographes*; (3) to study the morphological characters of the *I*. ser. *Sibiricae* species; and (4) to compare the results of molecular and morphological studies to determining the taxonomic composition of *I.* subser. *Chrysographes*.

## 2. Materials and Methods

### 2.1. Plant Samples, DNA Extraction, and Sequencing

**Figure 2:**
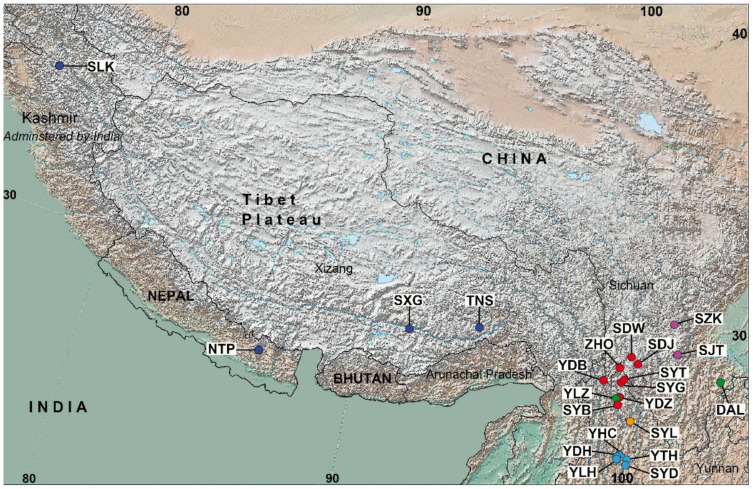
Map of *Iris* subser. *Chrysographes* localities. Codes correspond to entries in Table 1; cultivated plants (K6007, RUB, and K389) are not mapped. Circles represent localities: dark blue, *I. clarkei*; red, *I. bulleyana*; light purple, *I. chrysographes*; orange, *I. forrestii*; green, *I. wilsonii*; blue, *I. delavayi*.

A total of 25 fully verified samples representing *I.* subser. *Chrysographes* were used for the molecular analyses. All samples were taken from living collections or herbarium specimens, of which three were of unknown geographical origin and the others were from 22 localities in southwestern China (Yunnan, Sichuan, and Xizang provinces), India, and Nepal (Figure 2). The complete list of samples, including their origin and voucher information, is provided in Table 1.

The methods for DNA extraction, amplification, and direct sequencing of four non-coding cpDNA regions (*trnS*–*trnG*, *trnL–trnF*, *rps4–trnS*^GGA^, and *psbA*–*trnH*) have been described elsewhere [32,37]. Forward and reverse sequences for each region were determined on a genetic analyzer ABI 3130 (Applied Biosystems, Bedford, MA, USA) at the Joint Center of Biotechnology and Gene Engineering, the Federal Scientific Center of the East Asia Terrestrial Biodiversity (Vladivostok, Russia), and assembled using the Staden Package, version 1.4 [38]. The sequences previously obtained for *I.* *sibirica* [1] were also included in the analyses. In phylogenetic analyses, we also used the sequences previously published for representatives of three series of *I*. sect. *Limniris* [33,34]. These are (1) *I. laevigata* Fisch., *I. ensata* Thunb., and *I. pseudacorus* L. from *I*. ser. *Laevigatae* (Diels) G.H.M.Lawr.; (2) *I. lactea* Pall., *I. oxypetala* Bunge, and *I. tibetica* (Dykes) Bolt. from *I*. ser. *Lacteae* Doronkin; and (3) *I. uniflora* Pall. ex Link from *I*. ser. *Ruthenicae* (Diels) G.H.M.Lawr. In addition, *I. dichotoma* Pall. from *I.* subgen. *Pardanthopsis* (Hance) Baker was used as outgroup. The sequences obtained were deposited in the European Nucleotide Archive database. The accession numbers for all the sequences used are listed in Table 1.

### 2.2. Sequence Alignment and Phylogenetic Analyses

The sequences of each cpDNA region were aligned manually in SeaView, version 4 [39], and concatenated for each specimen. We included indels and length variation in mononucleotide repeats in the dataset because repeatability tests allowed excluding PCR errors. The haplotypes were identified based on combined DNA sequences using DnaSP, version 5 [40]. A network of haplotypes was constructed using Network, version 4.6 [41], with treating each deletion/insertion, regardless of size, as a single mutational event and using the MJ algorithm with default settings.

Phylogenetic analyses were conducted using the ML and MP methods as implemented in PAUP, version 4.0 b10 [42]. Bayesian analysis was performed using MrBayes, version 3.2.2 [43] via the CIPRES portal [44]. The dataset was composed of sequences from the *I*. subser. *Chrysographes* specimens, haplotypes H1–H8 of *I.* subser. *Sibiricae* [1], and sequences of species from three other series of *I.* sect. *Limniris* (i.e., *Laevigatae*, *Lacteae*, and *Ruthenicae*) and *I. dichotoma* as outgroup. For the MP analysis, gaps were coded according to the simple indel coding procedure [45] as implemented in FastGap, version 1.2 [46]. Optimal trees were found using a heuristic search with TBR branch swapping and the MulTrees option in effect. For ML and BI analyses, the GTR + I + G model was selected according to the Akaike information criterion using Modeltest, version 3.6 [47]. ML heuristic searches were done using the resulting model settings, 100 replicates of random sequence addition, TBR branch swapping, and MULTrees option on. In BI, using the default prior settings, two parallel MCMC runs were carried out for 10 million generations, sampling every 1000 generations for a total of 10,000 samples. Convergence of the two chains was assessed, and PP were calculated from the trees sampled during the stationary phase. The robustness of nodes in ML and MP trees was tested using bootstrap with 1000 replicates (BP).

The degrees of divergence between the groups identified in the MJ and phylogenetic analyses were calculated based on nucleotide substitutions using DnaSP. The distribution of genetic variation within and among these groups and *F*_ST_ among them was determined by AMOVA as implemented in Arlequin, version 3.5 [48]. The significance of the variance components and genetic distances were tested using 1000 random permutations.

### 2.3. Morphological Data

The main taxonomic works dealing with *I.* subser. *Chrysographes* were consulted [4,10,11,13,14,18]. In order to clarify morphological characters of the species and compile the morphological key, the herbarium specimens deposited at BM, E, K, and LE (herbarium codes according to Thiers [36]), including the type material of the names studied, were examined personally by the authors [19]. In addition, the specimens have been searched through high-resolution images available in virtual herbaria: BNU, CDBI, HITBC, HNWP, IBK, IBSC, IMC, IMDY, JIU, KUN, LBG, NAS, PEM, Shangri-la Alpine Botanic Garden (as SABG), SZ, WCSBG and XBGH (https://www.cvh.ac.cn/; accessed on 15 September 2021), BM (https://data.nhm.ac.uk/dataset/collection-specimens; accessed on 15 September 2021), E (https://data.rbge.org.uk/search/herbarium/; accessed on 15 September 2021), K (http://apps.kew.org/herbcat/navigator.do; accessed on 15 September 2021), AMD, L and U (http://bioportal.naturalis.nl/; accessed on 15 September 2021), P (https://science.mnhn.fr/institution/mnhn/collection/p/item/search; accessed on 15 September 2021), and PE (http://pe.ibcas.ac.cn/en/; accessed on 15 September 2021). For the morphological study of the *I.* ser. *Sibiricae* species, sixteen descriptive characters were selected based on studies of the dedicated literature and herbarium specimens (Table 2 and Table S1, Figure 1a–c). Since the collected data were used to identify morphological distinctions in the entire series studied, we, hence, incorporated the detailed data on *I. sibirica* from recent work [1].

In a total, 540 specimens (see Annex 1) of well-developed plants in flowering and fruiting stages, collected from Bhutan, China, India, and Nepal, we examined based on the qualitative and quantitative morphological characters useful to distinguish species. The herbarium specimens were identified on the basis of our own experience in dealing with this group. The quantitative characters were measured using AxioVision, version 4.8 (Carl Zeiss, Germany).

### 2.4. Morphometric Analysis

Our morphometric analysis of *I. bulleyana*, *I. chrysographes*, *I. delavayi*, and *I. forrestii* was based on eight quantitative (BL, CL, LL, LW, NC, NF, PL, and SH) and one qualitative (IS) characters (Table 2). As the dataset of *I. delavayi* was limited to 13 individuals, for statistical analysis, we randomly selected 13 samples for each species from the initial dataset by using the built-in resample function of the R free software for statistical analysis [49], version 4.1 [50]. The dataset was analyzed by using one-way ANOVA. Differences were considered statistically significant at a *p*-value < 0.05. After a multiple statistical testing was performed, the calculated *p*-values were adjusted using the procedure proposed by Benjamini and Hochberg [51]. To test one-way ANOVA assumptions, the Shapiro–Wilk’s test for normality of distribution [50] and Levene’s test for equality of variances [52] were used. If an ANOVA showed a statistically significant difference among species, then subsequent pairwise comparisons were made using the Tukey’s post-hoc test [53]. Inequality of variance was taken into account by using the heteroscedastic consistent covariance estimation provided in the R add-on package “sandwich”, version 2.3.0 [54,55]. Analysis of the countable characters (NF and NC) was done by the Poisson regression using the respective R built-in function [50].

We conducted the PCA analysis [56] to visualize the distribution of species over the space of quantitative multivariate data and to assess their delimitation. The characters were considered taxonomically useful when overlap was equal to or lower than a threshold of 25% [57]. The PCA analysis was performed using the built-in function *prcomp*, and the results of the analysis were extracted and visualized using the respective functions of the *factoextra* R package [58]. CV (%) was calculated for the six quantitative characters (Table S2). Values of CV were classified in four categories: minor variation (0–10), little variation (11–20), average variation (21–40), and high variation (41–60). All statistical analyses were performed using the R Statistical Software, version 4.1 [50].

### 2.5. Taxonomy and Distribution

Here, the conservative taxonomy of *Iris* is used [2,6,7,10,13,14,22,24]. The types of the *I*. subser. *Chrysographes* names were selected in a recent nomenclatural study [19]. For the taxonomy, the *Shenzhen Code* [59] was consulted. In the case of disagreement on the infraspecific rank at which a name should be accepted, we followed Brummitt [60]. In the Taxonomic treatment section (see below), we extracted the information on distribution of the accepted taxa from the herbarium specimens. We also consulted the information provided in References [4,10,11,13,14,18,20,22,61], which are commonly recognized as taxonomically reliable sources.

## 3. Results

### 3.1. Genetic Divergence of Chloroplast Non-Coding Sequences within Iris Subser. Chrysographes

Among the 25 specimens of *I.* subser. *Chrysographes*, 12 haplotypes (C1–C12; see Table 1) were identified on the basis of polymorphic sites detected across 3704 aligned positions of four cpDNA regions. Four of these haplotypes (C1, C2, C7, and C9) were found in several localities, sometimes geographically very distant from each other (e.g., C9 in SLK and TNS from India and Nepal, respectively), while the others were unique, i.e., found in a single locality.

**Figure 3:**
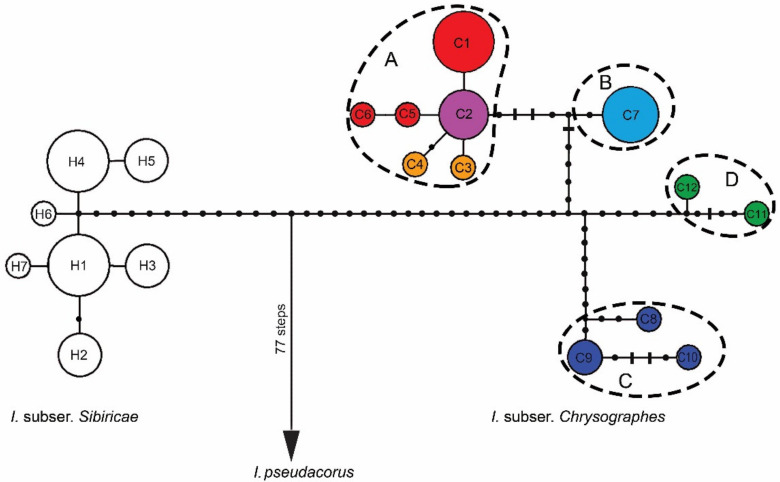
Median-joining networks inferred from combined sequences of the *trn*S–*trn*G, *trn*L*–trn*F, *rps*4*–trn*S^GGA^, and *psb*A–*trn*H regions showing the relationships among cpDNA haplotypes of the *Iris* ser. *Sibiricae* species and *I. pseudacorus* as outgroup. Each circle indicates a haplotype, with the size of the circle proportional to the number of populations where this haplotype is found. The colors of circles indicate the affiliation of haplotype: white, *I. sibirica*; red, *I. bulleyana*; light purple, *I. chrysographes*; orange, *I. forrestii*; blue, *I. delavayi*; green, *I. wilsonii*; dark blue, *I. clarkei.* Black dots indicate intermediate haplotypes not observed in the samples; short bars indicate indels; the haplotypes outlined by dashed lines represent groups A–D within *I*. subser. *Chrysographes*. For haplotype codes, see Table 1.

The relationships between haplotypes found in representatives of *I.* ser. *Sibiricae* are shown in Figure 3. All haplotypes of *I.* ser. *Sibiricae* are connected into a single network without loops and derived from the same extinct ancestral haplotype related through many mutational steps with the haplotype of *I*. *pseudacorus*. Haplotypes of *I.* subser. *Chrysographes* (C1–C12) are separated by 29 mutational steps from haplotypes of *I.* subser. *Sibiricae* (H1–H8) closely related to each other. The more heterogeneous haplotypes of *I.* subser. *Chrysographes* form four groups (A–D) separated from each other by multiple (from 6 to 15) mutational steps (Figure 3). Haplogroup A contains six closely related haplotypes (C1–C6), most of which differ by only one mutational step from C2, forming a star-like structure indicative of a rapid range expansion in the past. Haplotypes of this group are found in samples of *I. bulleyana* (C1, C5, and C6), as well as in samples of *I. chrysographes* (C2) and *I. forrestii* (C3 and C4).

Haplogroup B includes one haplotype C7, which was found in samples from different localities near the Cang Mountains (Yunnan Province, China), and is separated from haplogroup A by 7 mutational steps. Haplogroups C and D are separated from each other by 11 mutational steps and from other two haplogroups (A and B) by 12–14 mutational steps including 9-bp insertion in the *trnL*–*trnF* region. Haplotypes of C group were found in the specimens from the Xizang Province, China (C8 in SXG and C9 in TNS), and the Eastern Himalayas (C9 and C10 in SLK and NTP, respectively). Haplotypes C11 and C12 of haplogroup D were found in the *I. wilsonii* specimens: YLZ from Shangri-La (formerly known as Zhongdian; Yunnan, China) and DAL from the Daliang Mountains (Sichuan, China). The pairwise *F*_ST_ values between four haplogroups varied from 0.609 to 0.929 (*p* < 0.05), and *K*_S_ varied from 0.00374 to 0.00447.

**Figure 4:**
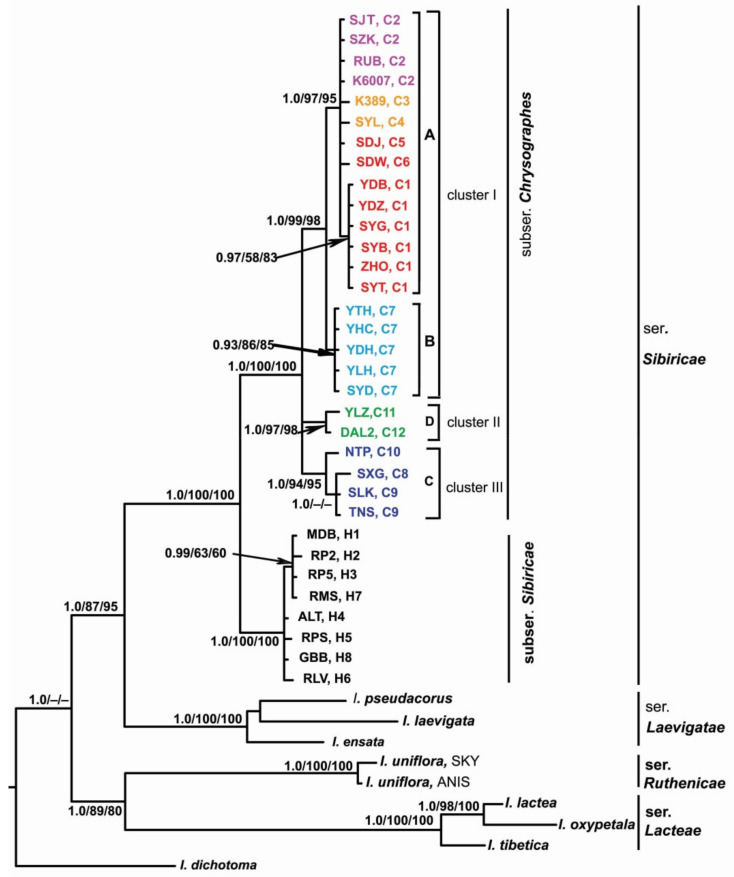
The Bayesian majority rule consensus tree of the *Iris* ser. *Sibiricae* samples inferred from combined *trnS*–*trnG*, *trnL*–*trnF*, *rps4*–*trnS*^GGA^, and *psbA*–*trnH* chloroplast data. The numbers above the branches are Bayesian posterior probabilities (PP > 0.9) and bootstrap values (>50%) for MP and ML analyses. The haplotype and locality codes correspond to those in Table 1. A, B, C, and D indicate the haplogroups in Figure 3. Color indicates the affiliation of haplotype: light purple, *I. chrysographes*; orange, *I. forrestii*; red, *I. bulleyana*; blue, *I. delavayi*; green, *I. wilsonii*; dark blue, *I. clarkei*.

In all the phylogenetic analyses, *Iris* accessions were distributed with a robust support (PP 1.0, BP 100 and 100%) in accordance with their affiliation to the corresponding series of *I*. sect. *Limniris* (Figure 4). *Iris* ser. *Sibiricae* was resolved as a monophyletic group (PP 1.00, BP 100 and 100%) consisting of two strongly supported sister clades corresponding to *I.* subser. *Sibiricae* (PP 1.0, BP 100 and 100%) and *I.* subser. *Chrysographes* (PP 1.0, BP 100 and 100%) that are recognized in this group. The pairwise *F*_ST_ between these subseries was 0.825 (*p* = 0.00001), and *K*_S_ between them was 0.00892. Within clade *I.* subser. *Chrysographes*, there was a polytomy of three monophyletic clusters, with the divergence between them varying from 0.00408 to 0.00440 (Table S3). In cluster I (PP 1.0, BP 99 and 98%), two sister groups, A and B, were resolved with a support of PP 1.0, BP 97 and 95% and PP 0.93, BP 86 and 85%, respectively. These groups corresponded to haplogroups A and B revealed by the MJ-network analysis (Figure 3). The pairwise *F*_ST_ between these haplogroups was 0.86 (*p* = 0.00001), and *K*_S_ between them was 0.00153. The nucleotide divergence between the sequences of species comprising haplogroup A (*I. bulleyana*, *I. forrestii*, and *I. chrysographes*, hereinafter referred to as the *bulleyana* group) varied from 0.00023 to 0.00063 (Table S3). The pairwise *F*_ST_ values between them varied from 0.385 to 0.419 and were not significant (*p* > 0.05). No nucleotide substitutions or indels differentiating these species were revealed. The sequence divergence between each of these species and *I. delavayi* (from haplogroup B, Cluster I) was higher and varied from 0.00133 to 0.00173. Clusters II and III were consistent with haplogroups C and D, which was revealed by the MJ-network analysis (Figure 3).

### 3.2. Morphological Study

To evaluate the taxonomic significance of the molecular results, we performed a morphological study of the *I.* ser. *Sibiricae* species. The main diagnostic characters that allow distinguishing between the *I.* ser. *Sibiricae* species are summarized in Table 3. Morphologically, the *I*. subser. *Chrysographes* species are distinguished from *I. sibirica* by their longer bracts (most commonly more than 6 cm in length), which are also green at blooming (dry in *I. sibirica*), by the 1–2.2 cm long perianth tube (not longer than 0.5 cm in *I. sibirica*), and by the slightly veined ornamentation of the falls (strongly veined in *I. sibirica*; see Figure 1).

The results have shown as follows (Table 3): (1) *I. clarkei* is the only species in *I.* ser. *Sibiricae* with solid flowering stems (the rest of the species have hollow stems), which are 1–2-branched; (2) *I. delavayi* is the tallest species, having 1-branched flowering stems up to 114 cm long, which are usually higher than basal leaves; (3) *I. forrestii* and *I. wilsonii*, which are the only two yellow-flowered species, appear quite similar, with, however, *I. wilsonii* being generally more vigorous, having elongated cauline leaves up to 40 cm long (in *I. forrestii*, not longer than 25 cm), flowering stems of about the same length as basal leaves, and pale yellow flowers born on elongated pedicels up to 11 cm long (in *I. forrestii*, flowers are clear yellow with brownish-purple lines on the haft, and pedicels not exceeding 8 cm in length); and (4) *I. bulleyana*, *I. chrysographes*, and *I. forrestii* are morphologically most closely related, while differing mainly in the color of flowers.

Eigenvalues of the measurable morphological characters (principal components), which were essential to the observed variations between the taxa, are given in Table S2. Variables CL, LL, LW, NC, NF, and SH had the largest share in the separation of the species of the *bulleyana* group and *I. delavayi*, the closely allied species according to our molecular data. The cumulative percentage of the explained variance was 68.74%. A further analysis showed a statistically significant difference in BL and PL between *I*. *delavayi* on the one hand, and between *I*. *bulleyana* and *I*. *forrestii* on the other (*p* = 0.0012 and *p* < 0.0001, respectively). Between the species of the *bulleyana* group, no statistically significant difference was found in BL, CL, LL, NC, NF, and PL. A significant difference in LW and SH was found in the pair *I. forrestii* and *I. chrysographes*; the other pairs of species in the *bulleyana* group did not show any difference.

**Figure 5:**
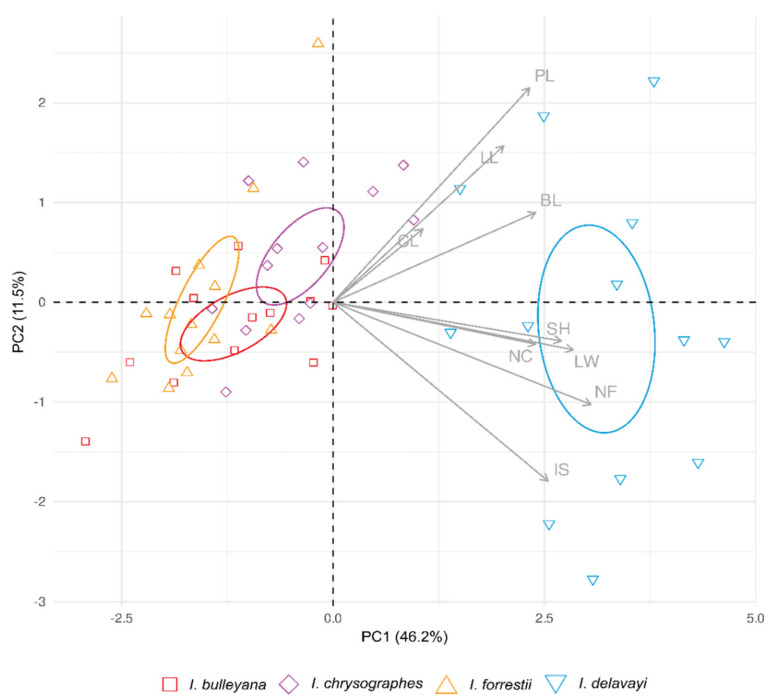
Principal component analysis (PCA) of morphological characters. PCA scatter-plot of the first two principal components based on 9 characters for 52 studied specimens. For character codes, refer to Table 2. The ellipses outline the confidence regions for the species with the respective color.

The PCA of the quantitative characters of the estimated variance component for all the samples gave values of 46.2% and 11.5%, respectively, for the first two principal components (Figure 5). Two characters, NF and PL, displayed the highest correlations with the first (NF, *r* = 0.85) and the second axis (PL, *r* = 0.60); the third one, CL, highly influenced the third axis (CL, *r* = −0.83). In the PCA scatter-plot of all the individuals in the plane defined by the first two principal components (Figure 5), the studied specimens grouped together in accordance with their taxonomic affiliation, creating two separate groups. The first one included the species of the *bulleyana* group, and the second one included *I. delavayi* (Figure 5). An examination of the biplot (Figure 5) from first two principal components revealed a partial overlapping of *I. bulleyana*, *I. chrysographes*, and *I. forrestii* and also their significant morphological similarity. The separation of *I. delavayi* from the other three taxa was defined by the first two principal components. Thus, having likely different average values of some morphometric characters caused by environmental conditions and interspecific trait variability, *I.* *bulleyana*, *I. chrysographes*, and *I. forrestii* can still be considered as indistinguishable in a generalized (PCA) factor space. Therefore, the result of PCA proved to be the same as that of the molecular study, and it was sufficient to supplement molecular evidence.

## 4. Discussion

Based on the sequencing of cpDNA regions for the samples from different localities within the *I*. ser. *Sibiricae* distribution range, our study confirms the monophyly of two main divergent lineages and a sister relationship between them. Such a pattern is generally consistent with results of several phylogenetic studies [6,24,62] and supports the splitting of *I*. ser. *Sibiricae* into two subseries, as it was previously suggested on the basis of chromosome numbers, morphology, and distribution (e.g., References [5,11]). The first lineage contains haplotypes of all samples from the northern part of the range, where one species (*I. sibirica*) is distributed [1], and the second one contains haplotypes of samples from the southern part of the range, where species of *I.* ser. *Sibiricae* with 2n = 40 chromosomes are distributed [11,13,14]. The high levels of genetic differentiation and nucleotide divergence of cpDNA between these lineages indicate a deep genetic split between them, which may suggest a long independent evolutionary history of species from the two subseries.

Recently, six taxa at the species rank have been assigned to *I.* subser. *Chrysographes* [19]. Based on the cpDNA region sequence analyses, we revealed four distinct genetic lineages (Figure 3 and Figure 4). The values of nucleotide sequence divergence between these lineages (Table S3) are comparable with the divergence between other species in *Iris* [32,34] and between closely related species in other genera (e.g., References [63,64]). The lowest value was found between haplogroups A and B (Table S3) forming a single cluster (I) in the phylogenetic analyses (Figure 4), which is indicative of close genetic relationships between the species constituting them. In our study, we could not distinguish genetically between these three taxa with minimally divergent haplotypes lacking species-specific markers and forming a star-like structure in the network, which indicates no deep phylogenetic split between them and is consistent with the rapid range expansion. The species forming haplogroup A, i.e., the *bulleyana* group, can easily interbreed with each other during cultivation and in the wild and, thus, lose their identity [4,14].

Grey-Wilson [4,11] believed that *I. clarkei* might perhaps best be placed in a separate group of its own. On the contrary, our molecular data showed that both *I. clarkei* and *I. wilsonii* are placed in the monophyletic clade of *I.* subser. *Chrysographes* as distinct lineages. Dykes [13] suggested that *I. forrestii* may roughly be described as a dwarf *I. wilsonii*. It should be noted that, despite the fact that the overall distributions of *I. forrestii* and *I. wilsonii* appear to overlap, there is no indication that the two species grow together in the wild and, therefore, their natural hybridization is likely to be ruled out [11]. Moreover, *I. forrestii* blooms about two weeks earlier than *I. wilsonii* [65]. Our results suggest a phylogenetic affinity of *I. wilsonii* with *I.* *clarkei*. In addition, the molecular data shows that *I. delavayi* is the closest species to the species of the *bulleyana* group and appears as a sister taxon in the same cluster with them. This species is distinct in morphology (Table 3), being, however, more genetically similar to *I. chrysographes* than to any other of the *I.* subser. *Chrysographes* species. Thus, our analyses support the recognition of *I.* *clarkei*, *I.* *delavayi*, and *I.* *wilsonii* as distinct species.

Traditionally, *I. clarkei* is regarded as unique and holding an isolated position within *I.* ser. *Sibiricae*, as it has a solid flowering stem (e.g., References [10,11,13,14]). However, according to some authors [20,66] and the herbarium specimens (e.g., L3912484; see Annex 1), the flowering stem in *I. clarkei* is considered to be solid with a small central hollow. We assume that, generally or in some cases, the flowering stem in *I. clarkei* is not completely solid, with the central part of the stem filled with a broad expanse of pith. Similarly, it is generally accepted that *I. wilsonii* has an unbranched flowering stem with a terminal inflorescence of two flowers, as we observed when examining the herbarium specimens. Contrary to this, we found specimens of *I. wilsonii* heaving 1-branched flowering stems. These plants were collected in Zhaojue County, Sichuan Province (the gatherings “*W. Sun 15*” and “*Sichuan Vegetation Team* *12818*”; see Annex 1).

There is a high degree of morphological similarity between specimens belonging to the *bulleyana* group, which probably results from their close relationship, as indicated by many researchers. As pointed out by Stapf [67] and Dykes [13,22,68], the author of the *bulleyana* group taxa, *I. chrysographes* is closely allied to *I. forrestii*, while differing in color of flowers and in habitats, and *I. bulleyana* also strongly resembles *I. forrestii.* According to Noltie [10,21], the evidence of the variability observed in the northwestern Yunnan Province in 1993 provides much support to the idea of treating *I. chrysographes* and *I. bulleyana* as forms of the same species differing mainly in the flower color, and our results suggest that this assumption is reasonable. In fact, it was confirmed on the basis of the morphological results that *I. bulleyana*, *I. chrysographes*, and *I. forrestii* have considerable morphological variation, and, even though the studied specimens exhibit continuity of morphological characters, they, however, differ in flower color (Table 3). In addition, the morphometric analysis showed one overlapping group in which the specimens of the *bulleyana* group were indistinctly separated from each other and formed a single aggregation (Figure 5).

In addition, our examination of the herbarium specimens showed (see Annex 1) that the species of the *bulleyana* group have the same distribution in China. In the Yunnan Province, these are common in Dali Bai Autonomous Prefecture, Diqing Tibetan Autonomous Prefecture (Haba Snow Mountain, Shangri-La county-level city), the Lijiang prefecture-level city (Yulong Naxi Autonomous County, including *I. bulleyana* f. *alba*), and Nujiang Lisu Autonomous Prefecture. In the Sichuan Province, these are common in Garzê Tibetan Autonomous Prefecture (Jiulong County), and Liangshan Yi Autonomous Prefecture (Muli and Yanyuan counties); additionally, *I. bulleyana* and *I. chrysographes* occur in Ngawa Tibetan and Qiang Autonomous Prefecture (Mianning County). Moreover, as was reported in previous works [11,14] and is confirmed in the present study, *I. bulleyana* is sometimes found in associations with *I. chrysographes* and *I. forrestii* (e.g., the gathering “*C.W. Wang 63721*”; see Annex 1) or in mixed populations of *I. bulleyana* and *I. chrysographes* (e.g., the gatherings “*G. Forrest 26948*”, “*C.W. Wang 67662*”, “*T.T. Yü 11650*”, “*G. Forrest 25043*”, “*Tibet Chinese Herbal Medicine Survey Team 3137* & *4024*”, “*Qinghai-Tibet team 751074*”, and “*Z. Liu* 4726”).

Thus, *I. bulleyana*, *I. chrysographes*, and *I. forrestii* do not differ in characters that are significant for taxonomic species differentiation within the genus, and the molecular data and morphometric characters selected here are sufficient to confirm this assumption. In such a situation, combining the critical taxa into a single species seems to be appropriate, and many researchers have adopted this approach (e.g., Reference [69]). For this reason, further determination of the three species with their overlapping distribution ranges is obviously not justified, and, therefore, we suggest reconsidering their taxonomic status.

## 5. Taxonomic Treatment

In the present study, we confirm that *I.* ser. *Sibiricae* is divided into two groups, the autonymic subseries with a single species *I. sibirica* [1] and *I.* subser. *Chrysographes*. As a consequence of the present work, we consider form rank to be the most suitable option for *I. bulleyana*, *I. chrysographes*, and *I. forrestii*. The earlier described names, *I. bulleyana* and *I. forrestii*, were simultaneously published by Dykes [70], both with equal priorities until now. Here, we combine these taxa and establish priority of *I. bulleyana* over *I. forrestii*, the other competing name (see Art. 11.5, Note 3 of the ICN).

### 5.1. The List of Taxa

A list of the taxa within *I.* subser. *Chrysographes* accepted in the present work and information on species distributions, habitats, and types is provided below.

(1) ***Iris clarkei*** Baker ex Hook.f., Fl. Brit. India 6(18): 275, 1892 ≡ *Limniris clarkei* (Baker ex Hook.f.) Rodion., Bot. Zhurn. 92(4): 551, 2007.—Lectotype (designated by Boltenkov [19] (p. 290)): [India] Sikkim Himal., [fl.], 1848, [*Hooker*] *s.n.* (K000098495!, specimens at the flowering stage).—http://specimens.kew.org/herbarium/K000098495 (accessed on 15 September 2021).

*Distribution and habitat*—This species has a more westerly distribution and is native to the Central and Eastern Himalayas, particularly to northeastern India (the states of Manipur, Sikkim, and West Bengal and the Ladakh union territory), central and eastern Nepal, the Haa and Paro valleys in western Bhutan [71], and northern Myanmar [72]. In southwestern China, it is distributed in the southeastern Xizang Province (the Nyingchi and Shigatse prefecture-level cities) and northwestern Yunnan Province (Nujiang Lisu Autonomous Prefecture). It grows commonly in shady places in marshes, wet meadows, woodland margins, and beside streams and lakes at elevations of 2300–4300 m.

(2) ***Iris delavayi*** Micheli, Rev. Hort. 67: 398, 1895 ≡ *Limniris delavayi* (Micheli) Rodion., Bot. Zhurn. 92(4): 551, 2007.—Lectotype (designated by Boltenkov [19] (p. 290)): [illustration] “*Iris delavayi*” in Micheli [73] (p. 399, f. 128).—https://www.biodiversitylibrary.org/item/197620#page/437/mode/1up (accessed on 15 September 2021).

*Distribution and habitat*—This species is distributed in the Chumbi Valley (Yadong County, southeastern Xizang Province, China) and near the Cang Mountains in the northwestern Yunnan Province, China (Dali City and Yangbi Yi Autonomous County). In southwestern China, *I. delavayi* also occurs in the Guizhou and Sichuan provinces [10]; however, we could not confirm its distribution there with any reliable herbarium specimens and, therefore, it requires verification. The populations of this semi-aquatic species are severely fragmented in distribution. They generally grow in damp places along ditches and streams, in wet or marshy mountain meadows, and swampy places at elevations of 2300–3400 m.

(3) ***Iris wilsonii*** C.H.Wright, Bull. Misc. Inform. Kew 26(8): 321, 1907 ≡ *Limniris wilsonii* (C.H.Wright) Rodion., Bot. Zhurn. 92(4): 551, 2007.—Lectotype (designated by Boltenkov [19] (p. 292)): [Specimen from a cultivated plant], China, Wilson, Kew, 26 June 1907, [fl.], *s. coll. 1164a* (K000499094!).—http://specimens.kew.org/herbarium/K000499094 (accessed on 15 September 2021).

*Distribution and habitat*—It is an endemic to central and southwestern China and has a more easterly distribution: the southeastern Gansu Province (Hui County), the southern Shaanxi Province (Yang, Fuping and Ningshan counties, and the Baoji prefecture-level city), the northern and southern Sichuan Province (Leibo, Mianning, Meigu, Yuexi, and Zhaojue counties, and Ebian Yi Autonomous County), the northern and southern Chongqing municipality (Chengkou, Wushan, and Wuxi counties, and Nanchuan District), and the western Hubei Province (Badong and Fang counties, and Shennongjia Forestry District). It is often found on hillsides, wet meadows, forest edges, along riversides and streams at elevations of 1600–3600 m and, apparently rarely, above.

(4) ***Iris bulleyana*** Dykes, Gard. Chron., ser. 3, 47: 418, 1910 ≡ *Limniris bulleyana* (Dykes) Rodion., Bot. Zhurn. 92(4): 551, 2007.—Lectotype (designated by Boltenkov [19] (p. 292)): [China], Fl. blue, Marais, sur le Yu Kia Ngan, au dessus de Pon Man tsen, á 2800 m, [fl.], 3 July 1888, *J.M. Delavay 4808* (P02159142 [digital image!]).—http://coldb.mnhn.fr/catalognumber/mnhn/p/p02159142 (accessed on 15 September 2021).

*Distribution and habitat*—This species is endemic to the Hengduan Mountains in southwestern China, where it is very common. It grows in moist areas among grasses on hillsides or forest edges, in meadows, pastures, and beside streams at elevations of 1800–4800 m.

(4.1) ***Iris bulleyana f. bulleyana***

*Distribution*—In China, it occurs in the northwestern Yunnan Province: Nujiang Lisu Autonomous Prefecture (Gongshan Dulong and Nu Autonomous County), northern Dali Bai Autonomous Prefecture (Jianchuan County), Diqing Tibetan Autonomous Prefecture (Deqin and Weixi Lisu Autonomous counties, and the Shangri-La county-level city), the Lijiang prefecture-level city (Yulong Naxi Autonomous County); in the southwestern Sichuan Province: Garzê Tibetan Autonomous Prefecture (Jiulong and Xiangcheng counties, and the Kangding county-level city), Liangshan Yi Autonomous Prefecture (Muli and Yanyuan counties), Ngawa Tibetan and Qiang Autonomous Prefecture (Zoigê and Mianning counties); and in southeastern Xizang Province: the Nyingchi prefecture-level city (Mainling and Zayü counties, Bayi District). In addition, it occurs in northern Myanmar (Kachin State).

(4.2) ***Iris bulleyana*** f. ***forrestii*** (Dykes) Bolt., ***comb. et stat. nov.*** ≡ *I. forrestii* Dykes, Gard. Chron., ser. 3, 47: 418, 1910 (basionym) ≡ *Limniris forrestii* (Dykes) Rodion., Bot. Zhurn. 92(4): 551, 2007.—Lectotype (designated by Boltenkov [19] (p. 292)): [China] NW Yunnan, open mountain meadows on the eastern flank of the Lichiang Range, lat. 27°30′ N, alt. 11–12,000 ft., [fl.], June 1906, *Forrest 2426* (E00381810!).—https://data.rbge.org.uk/herb/E00381810 (accessed on 15 September 2021).

*Distribution*—Northwestern Yunnan Province: the Baoshan and Lijiang (Yulong Naxi Autonomous County) prefecture-level cities, northern Dali Bai Autonomous Prefecture (Heqing County), Diqing Tibetan Autonomous Prefecture (Deqin County and the Shangri-La county-level city), Nujiang Lisu Autonomous Prefecture (Lanping Bai and Pumi Autonomous County, Gongshan Derung and Nu Autonomous County); the southwestern Sichuan Province: Garzê Tibetan Autonomous Prefecture (Jiulong County), Liangshan Yi Autonomous Prefecture (Muli and Yanyuan counties), and the Panzhihua prefecture-level city (Yanbian and Miyi counties).

(4.3) ***Iris bulleyana*** f. ***chrysographes*** (Dykes) Bolt., ***comb. et stat. nov.*** ≡ *I. chrysographes* Dykes, Gard. Chron., ser. 3, 49: 362, 1911 (basionym) ≡ *Limniris chrysographes* (Dykes) Rodion., Bot. Zhurn. 92(4): 551, 2007.—Lectotype (designated by Boltenkov [19] (p. 293)): [Sichuan Province, China] fl. violet, thickets, common, west of Kuan Hsien [Dujiangyan City], 7–11,000 ft., [fl.], June 1908, *E.H. Wilson 1304* (K000499091!)—http://specimens.kew.org/herbarium/K000499091 (accessed on 15 September 2021).

*Distribution*—Northwestern Yunnan Province: Nujiang Lisu Autonomous Prefecture (the Lushui county-level city, Lanping Bai and Pumi Autonomous County), Diqing Tibetan Autonomous Prefecture (Weixi Lisu Autonomous County and the Shangri-La county-level city), northern Dali Bai Autonomous Prefecture (Eryuan County), the Lijiang prefecture-level city (Yulong Naxi Autonomous County); Sichuan Province: Garzê Tibetan Autonomous Prefecture (Daocheng and Jiulong counties, and the Kangding county-level city), Ngawa Tibetan and Qiang Autonomous Prefecture (Mao, Mianning, and Wenchuan counties), Liangshan Yi Autonomous Prefecture (Meigu, Muli, and Yanyuan counties); the southeastern Xizang Province: the Nyingchi prefecture-level city (Mainling County and Bayi District).

(4.4) ***Iris bulleyana*** f. ***alba*** Y.T.Zhao, Acta Phytotax. Sin. 18(1): 54, 1980.—Holotype: [Yunnan Province, China], Atuntze, mt. Paima Shan [Baimang Xueshan (Snow Mountains)], hillside, 3510 m, [fl.], 6 July 1937, *T.T. Yü 8749* (KUN0360168 [digital image!]).—https://www.cvh.ac.cn/spms/detail.php?id=ffb63158 (accessed on 15 September 2021).

*Distribution*—This is the rarest representative of *I. bulleyana*, found in the Yunnan Province: the Lijiang prefecture-level city (Yulong Naxi Autonomous County) and Diqing Tibetan Autonomous Prefecture (Deqin County).

### 5.2. The Key

Below is a key to the *I.* subser. *Chrysographes* taxa recognized in the present study.

1. Flowering stem nearly solid, usually 1–2-branched in the upper part; standards strongly inclined … *Iris clarkei*

1. Flowering stem hollow, 1-branched or unbranched; standards nearly erect or obliquely spreading … 2

2. Flowering stem usually more than 100 cm tall and higher than rosette leaves, 1-branched; rosette leaves usually more than 1 cm wide, without obvious midribs; standards obliquely spreading … *Iris delavayi*

2. Flowering stem up to 73 cm tall, almost as long as rosette leaves, with terminal inflorescence of 1–2 flowers; rosette leaves with obvious midribs, usually less than 1.1 cm in width; standards nearly erect … 3

3. Pedicels usually elongated (up to 11 cm long); flowers pale yellow … *Iris wilsonii*

3. Rosette leaves without obvious midribs; pedicels not exceeding 9 cm in length; standards inclined at an angle of 45°; flowers vary in color … 4

4a. Flower color variable, from pale blue to mauve and violet; blades of falls with white or yellow mottled and striped pattern … *Iris bulleyana* f. *bulleyana*

4b. Flowers usually lemon-yellow … *Iris bulleyana* f. *forrestii*

4c. Flower color variable, from reddish violet to deep violet; blades of falls often with golden yellow stripes … *Iris bulleyana* f. *chrysographes*

4d. Flowers white … *Iris bulleyana* f. *alba*

## 6. Conclusions

Here, we, for the first time, resolve the phylogenetic relationships of the *I*. ser. *Sibiricae* species and confirm the monophyly of two divergent lineages, subseries, and their taxonomic statuses. Our preliminary analyses highlight the need for a comprehensive study of genetic and morphological divergence in order to clarify the taxonomy of *I*. subser. *Chrysographes*, better known to horticulturists as Sino-Siberians. We have obtained molecular evidence and revised the plant morphology and distribution. Our perhaps most important conclusion is that a revision of some of the species-level taxa is required. Once again, we have shown that the chloroplast markers *trnS*–*trnG*, *trnL*–*trnF*, *rps4*–*trnS*^GGA^, and *psbA*–*trnH* provide a reliable resolution of the species and are optimal molecular markers for identifying taxonomic and phylogenetic relationships within critical taxa of the genus *Iris*.

The four lineages of *I.* subser. *Chrysographes* correspond to four morphologically distinct, biogeographically congruent groups: *I. clarkei* in the western part of the range, *I. wilsonii* in the eastern part, and *I. delavayi* and a complex of *I. bulleyana*, *I. chrysographes*, and *I. forrestii* in the central part of the range. In addition, our data show that the morphological characters of *I. bulleyana*, *I. chrysographes*, and *I. forrestii* were within the range of variation of a single species to which they are assigned. A morphometric analysis based on nine morphological characters has not revealed any separation between the three taxa. In congruence with the molecular data, *I. bulleyana*, *I. chrysographes*, and *I. forrestii* show weak morphological differentiation and, thus, are better to be treated as color forms of the same species. Based on our present results, we accept *I.* *clarkei*, *I.* *delavayi*, and *I.* *wilsonii* in their traditional concepts and recognize the other three taxa as a single species. Therefore, we suggest two combinations, *I. bulleyana* f. *forrestii* and *I. bulleyana* f. *chrysographes*.

Molecular evidence obtained in this study contribute to the knowledge of the taxonomy of irises and their distribution in China. Thus, one of the goals set for the future is to produce a thoroughly verified, sufficiently sampled, and robust phylogenetic tree that would provide a basis for a revised phylogeny of *Iris* s.l. Furthermore, a thorough re-examination of some morphological characters is also needed, using a broader set of samples across the entire distribution ranges of the species considered, since we still lack adequate understanding of the flowering stem structure in *I. clarkei* and the flowering stem branching in *I. wilsonii*.

## Figures and Tables

**Table 1:** Sampled taxa with voucher information and GenBank accession numbers.

Code (Haplotype)	Locality (Voucher *)	GenBank Accession Numbers *trnH*–*psbA*/*rps*4–*trnS*/*trnS*–*trnG*/*trnL*–*trnF*
*I.* ser. *Sibiricae* subser. *Chrysographes*		
*I. bulleyana*		
ZHO (C1)	China, Yunnan, Zhongdian, *M.G. Pimenov* et al. *432* (MW)	LT627895/LT628011/LT628021/LT628001
SYB (C1)	China, Yunnan, Diqing, Beima Shan, *AGS Expedition 866* (E)	LT978551/LT981293/LT984443/LT984476
SYG (C1)	China, Yunnan, Zhongdian, Geza, *AGS Expedition 509* (E)	LT978550/LT981292/LT984442/LT984475
SYT (C1)	China, Yunnan, Zhongdian, Geza, Xiaoxue Shan, *AGS Expedition 1890* (E, cult.)	LT978549/LT981291/LT984441/LT984474
YDB (C1)	China, Yunnan, Bai Ma Shan, *B. Alden* et al. *1028* (E, cult.)	LR597324/LR597340/LR597356/LR597372
YDZ (C1)	China, Yunnan, Zhongdian, *E.J. Cowley 279* (Kew No. 1990-3337)	LR597325/LR597341/LR597357/LR597373
SDJ (C5)	China, Sichuan, Daocheng, *Sichuan Expedition 273* (E)	LR597326/LR597342/LR597358/LR597374
SDW (C6)	China, Sichuan, Daocheng, *D.E. Boufford* et al. *28151* (E, cult.)	LR597327/LR597343/LR597359/LR597375
*I. forrestii*		
SYL (C4)	China, Yunnan, Lijiang, Yulong Xueshan, *P. Cox* et al. *2633* (E, cult.)	LT978553/LT981295/LT984445/LT984478
K389 (C3)	Origin unknown (Kew No. 2015-389)	LR597332/LR597348/LR597364/LR597380
*I. chrysographes*		
SJT (C2)	China, Sichuan, Jiulong, *Sichuan Expedition 331* (E)	LR597328/LR597344/LR597360/LR597376
SZK (C2)	China, Sichuan, *Erskine* et al. *364* (Kew No. 1988-4993)	LR597329/LR597345/LR597361/LR597377
K6007 (C2)	Origin unknown (Kew No. 1969-6007)	LR597331/LR597347/LR597363/LR597379
RUB (C2)	Cultivar ”Rubella” (Kew No. 1949-59002)	LR597330/LR597346/LR597362/LR597378
*I. delavayi*		
SYD (C7)	China, Yunnan, Dali Xian, Yinglofen, *Sino-Amer. Bot. Expedition 959* (MHA)	LT978552/LT981294/LT984444/LT984477
YTH (C7)	China, Yunnan, Tsang Shan, Little Huadianba, *s.coll. 1561* (E, cult.)	LR597333/LR597349/LR597365/LR597381
YHC (C7)	China, Yunnan, Huadianba, Cang Shan Range, *Brickell & Leslie 12617* (Kew No. 1988-863)	LR597334/LR597350/LR597366/LR597382
YDH (C7)	China, Yunnan, Tsang Shan, Huadianba, *E.J*. *Cowley 1399* (Kew No. 1990-3528)	LR597335/LR597351/LR597367/LR597383
YLH (C7)	China, Yunnan, Tsang Shan, Little Huadianba, *E.J*. *Cowley 1561* (Kew No. 1990-3549)	LR597336/LR597352/LR597368/LR597384
*I. wilsonii*		
YLZ (C11)	China, Yunnan, Little Zhongdian, *E.J*. *Cowley 566* (Kew No. 1990-3457)	LR597339/LR597355/LR597371/LR597387
DAL2 (C12)	China, Sichuan, Daliang, *s.coll. 1229* (E)	*LT978548*/*LT981290*/*LT984440*/*LT984473*
*I. clarkei*		
SXG (C8)	China, Tibet, Gongbogyamda, *F.* *Ludlow* et al. *14065* (E, cult.)	LT978546/LT981288/LT984438/LT984471
TNS (C9)	China, Xizang, *Erskine* et al. *52* (Kew No. 1996-245)	LR597337/LR597353/LR597369/LR597385
SLK (C9)	Ladakh, Kargil, *C.A. Chadwell 82* (E, cult.)	LT978547/LT981289/LT984439/LT984472
NTP (C10)	Nepal, Trogsindho Pass, *E.F. Needham 674* (E, cult.)	LR597338/LR597354/LR597370/LR597386
*I.* ser. *Sibiricae* subser. *Sibiricae * *I. sibirica*		
(H1)	Mongolia, Dornod, Bayan-Uul, *Gubanov 550* (MW)	LT978556/LT981298/LT984448/LT984480
(H2)	Russia, Primorsky Krai, Khankaysky District, Il’inka, *Pshennikova s.n.* (VBGI)	LT978530/LT981272/LT984422/LT984455
(H3)	Russia, Primorsky Krai, Pokrovka, *Denisova & Talovskaya s.n.* (VBGI)	LT978532/LT981274/LT984424/LT984457
(H4)	Armenia, Lori, *Tamanyan* *& al. 07-1189* (ERE)	LT978527/LT981269/LT984419/LT984452
(H5)	Russia, Sebezhsky District, *Konechnaya s.n.* (LE)	LT978538/LT981280/LT984430/LT984463
(H6)	Russia, Leningrad Oblast, near Vyborg, *Boltenkov s.n.* (LE)	LT978545/LT981287/LT984437/LT984470
(H7)	Russia, Setun River, *Nasimovitch & Shchukin* *s.n.* (MHA)	LT978541/LT981283/LT984433/LT984466
(H8)	Georgia, Borjomi, *Merello s.n.* (LE)	LT978543/LT981285/LT984435/LT984468
**Outgroup specimens**		
*I.* ser. *Laevigatae*		
*I. ensata*	Russia, Primorsky Krai, Zarubino, *Boltenkov s.n.* (VBGI)	LT628002/LT628022/LT628012/LT627896
*I. laevigata*	Russia, Primorsky Krai, Roshchino, *Pshennikova s.n.* ( VBGI)	LT628003/LT628024/LT628013/LT627897
*I. pseudacorus*	Russia, Vladivostok, *Boltenkov s.n.* (VBGI)	LT628004/LT628025/LT628014/LT627898
*I.* ser. *Lacteae*		
*I. lactea*	Russia, Zabaykalsky Krai, Kharanor, *Chernova s.n.* (IRK)	LT627854/LN871708/LN871662/LN871625
*I. oxypetala*	China, Shaanxi, Suyde, *Kabanov s.n.* (LE)	LT627844/LT627950/LT627975/LT627911
*I.* *tibetica*	China, Qinghai, Xining to Ta Er, *Long* et al. *3* (E)	LT627893/LT627939/LT627998/LT627933
*I. ser. Ruthenicae*		
*I. uniflora*	Russia, Primorsky Krai, Anisimovka, *Orlovskaya s.n.* (VBGI)	LT627832/LN871684/LN871640/LN871604
*I. uniflora*	Russia, Zabaykalsky Krai, Kyrinsky District, *Vologdina s.n.* (VBGI)	LT628008/LT628029/LT628018/LT627902
*I.* subgen. *Pardanthopsis*		
*I.* *dichotoma*	Russia, Amur Oblast, *Baranova s.n.* (LE)	LT978555/LT981297/LT984447/LT984483

* Herbarium codes according to Thiers [36]. Accession numbers in italics are reported in References [1,34]. Cult., cultivated.

**Table 2:** Morphological characters analysed in the *Iris* ser. *Sibiricae* species.

No.	Character	Code *	Description/Remarks
1	Rosette leaf length	LL	Measured from the base to the apex of the longest central leaf in a rosette
2	Rosette leaf width	LW	Measured at the broadest part of the widest rosette leaf
3	Flowering stem height	SH	Measured from the base of flowering stem to the base of outer bract
4	Flowering stem structure	–	Internal structure of flowering stem according to the literature data
5	Flowering stem branching	IS	Classified as unbranched (designated as 1; see Table S1), 1-branched (2), and 2-branched (3) inflorescence
6	Number of flowers	NF	Flowers per stem
7	Number of cauline leaves	NC	Leaves on the flowering stem nodes
8	Cauline leaf length	CL	Measured from the base to the apex of the upper cauline leaf
9	Bract length	BL	Measured from the base to the apex of outer bract
10	Pedicel length	PL	Measured from the base of terminal head to the ovary base of the first blooming flower
11	Tube length	TL	Measured from the ovary apex to the base of falls
12	Flower color	–	The basic flower color according to the literature data and herbarium labels
13	Falls ornamentation	–	Markings (lines and spots) on blades of outer perianth segments (falls), according to the literature data and herbarium labels
14	Standards arrangement	–	The spatial arrangement of the inner perianth segments (standards), according to the literature data
15	Fruit length	FL	Obtained for the first fruit of terminal head from specimens at fruiting (designated as “[fr.]”; see Annex 1)
16	Fruit shape	–	Obtained from specimens at fruiting

* The codes are provided for the characters examined in the herbarium specimens.

**Table 3:** Morphological characteristics of the *Iris* ser. *Sibiricae* species.

Character	*I. sibirica* *	*I. clarkei*	*I. delavayi*	*I. wilsonii*	*I. forrestii*	*I. bulleyana*	*I. chrysographes*
Rosette leaf length	24–99	41–76	24–66	22–72	11–63	12–56	16–58
Rosette leaf width	0.2–1.1	0.8–1.4	0.6–1.5	0.4–1.1	0.2–0.9	0.2–1.1	0.2–1.1
Flowering stem height	22–99	17–84	21–114	17–70	4–58	5–73	9–68
Flowering stem structure	Hollow	Solid (narrow central hollow?)	Hollow	Hollow	Hollow	Hollow	Hollow
Flowering stem branching	0–1	0–2	1	0(1?)	0	0	0
Number of flowers	1–6	2–5	2–3	1–2(3?)	1–2	1–2	1–2
Number of cauline leaves	(0)1–2(3)	2–4	1–5	1–2	(0)1–2(3)	(0)1–2(3)	(0)1–2(3)
Cauline leaf length	4–25	7–13	7–19	10–39	6–25	6–23	7–25
Bract length	2–6(7)	6–9	6–10	6–11	3–8	4–13	5–13
Pedicel length	0.4–6	3–10	2–10	3–11	1.5–8	1.2–8	1.3–9
Tube length	≤0.5	1.1–1.8	1.1–1.8	1.1–1.9	1–1.8	1–2.1	1–2.2
Flower color	Blue to violet	Deep blue to violet blue	Light to dark purple-blue	Pale yellow	Lemon-yellow	Pale blue to mauve and violet	Reddish violet to deep violet
Falls ornamentation	White	Pale yellow at base; white	White at base	Purplish at base	Brownish-purple	White or yellow	Yellow
Standards arrangement	Erect	Strongly inclined	Obliquely spreading	Nearly erect	Inclined at 45°	Inclined at 45°	Inclined at 45°
Fruit length	1.5–7.7	3–6	4.5–6	2.5–5	2–5	2–7	2.8–7
Fruit shape	Oblong ellipsoidal	Oblong ellipsoidal	Oblong cylindrical	Ellipsoidal	Ellipsoidal	Ellipsoidal	Ellipsoidal

* According to Reference [1]. All measurements are expressed in centimeters. See supplementary raw data in Table S1 for more details. Descriptions of the characters and their codes are provided in Table 2; for illustrations, see Figure 1.

## Data Availability

Sequences resulting from this study are deposited in GenBank.

## Supplementary Materials

The following are available online at https://www.mdpi.com/article/10.3390/plants10112232/s1, Annex 1: Complete list of specimens examined in the morphological analysis, Table S1: Raw data of the morphological analysis, Table S2: The results of the variance analysis of the *Iris* subser. *Chrysographes* species, Table S3: Nucleotide divergence between groups identified by the MJ (four haplogroups) and phylogenetic analyses (three clusters) of *Iris* subser. *Chrysographes* from 25 localities, and also between *I. bulleyana*, *I. forrestii*, and *I. chrysographes* (haplogroup A) and *I. delavayi* (haplogroup B) as inferred from the cpDNA data.

## Abbreviations

The following abbreviations are used in this manuscript. ANOVA, Analysis of variance; BI, Bayesian inference analyses; BP, Bootstrap percentage; cpDNA, Chloroplast DNA; CV, Coefficient of variation; DNA, Deoxyribonucleic acid; *F*_ST_, Pairwise genetic distances; ICN, *International Code of Nomenclature for algae, fungi, and plants*; *K*_S_, Nucleotide sequence divergence; MCMC Markov chain Monte Carlo methods; MJ, Median joining algorithm; ML, Maximum likelihood method; MP, Maximum parsimony method; PCA, Principal component analysis; PCR, Polymerase chain reaction; PP, Bayesian posterior probabilities; RBGE, Royal Botanic Garden Edinburgh; TBR, Tree bisection-reconnection.
