# Supplementary material for: Taxonomic Composition of Iris Subser. Chrysographes (Iridaceae) Inferred from Chloroplast DNA and Morphological Analyses
Source: Plants (Basel). 2021 Oct 20;10(11):2232. doi: 10.3390/plants10112232 (PMC8621552; doi:10.3390/plants10112232)
Supplement: Supplementary file 1 [file plants-10-02232-s001.zip › Annex 1.pdf]

Annex 1. Complete list of specimens examined in the morphological analysis. Asterisk (*) indicates the specimens accompanied by labels in Chinese. The type material of the names cited according to Boltenkov [19].

Iris bulleyana Dykes.—**CHINA**. **Yunnan Province**: Marais, sur le Yu Kia Ngan, au dessus de Pon Man tsen, á 2800 m, [fl.], 3 July 1888, *J.M. Delavay 4808* (P02159142, neotype of *I. bulleyana*; P02159146, isoneotype); Chienchuan–Mekong divide [Jianchuan County], margins of bogs and marshy pasture, 7–8000 ft., [fl.], June 1923, *G. Forrest 23576* (PE00987651); s.loc., [fl.], s.d., *G. Forrest 29924* (PE00987614); s.loc., [fl.], s.d., *G. Forrest 30065* (PE00987615); [Lijiang], Eastern slope of Mount Dyinaloko, alpine meadow, 13000 ft., [fl.], [1923?], *J.F. Rock 9007* (KUN0360181); s.loc., [fl.], s.d., *K.M. Feng 30870* (IBSC0629794)*; Mo-ting shan, alpine meadows, 12500–13000 ft., [fr.], September 1932, *J.F. Rock 23361* (IBSC0628573 & NAS00554994); Wei-se Hsien [Weixi Lisu Autonomous County], in bamboo thicket, 3650 m, [fr.], 4 October 1934, *H.T. Tsai 59663* (PE00987618), ibid. *H.T. Tsai 59737* (PE00987619), ibid. 9 October 1934, *H.T. Tsai 59737* (KUN0360160 & SZ00043010); Wei-si Hsien, ravine side, 2300 m, [fl.], June 1935, *C.W. Wang 63721* (KUN0360164, PE00987620 & PE00987621); Wei-si Hsien, meadow, 3200 m, [fl.], August 1935, *C.W. Wang 67662* (NAS00554995 & PE00987647); A-tun-tze, grassy slope, 2700 m, [fr.], September 1935, *C.W. Wang 69964* (KUN0360161 & PE00987595; PE00987893 [has been identified as *I. delavayi*]); Deqin County, Nan Guoyao, hillside meadow, 4100 m, [fr.], 6 November 1937, *T.T. Yü 10631* (PE00987602–PE00987604); [Diqing Tibetan Autonomous Prefecture, Deqin County], Atuntze, Bai-ma-shan, 4100 m, [fr.], 15 November 1937, *T.T. Yü 10775* (PE00987596–PE00987600); Chungtien [Shangri-La], mt. grassy slope, 3300 m, [fr.], 16 November 1937, *T.T. Yü 10962* (PE00987637 & PE00987638; PE00987900 [has been identified as *I. delavayi*]); Chungtien, Lichiashica, ditch side, 3300 m, [fl.], 17 June 1937, *T.T. Yü 11650* (KUN0360165 & PE00987628–PE00987630); Atuntze, mt. Paima Shan, hillside, 3510 m, [fl.], 6 July 1937, *T.T. Yü 8749* (KUN0360168, holotype of *I. bulleyana* f. *alba*; PE00987662–PE00987664, isotypes); Tungchuling, Nankukung, mt. open slope, common, 3910 m, [fl.], 10 July 1937, *T.T. Yü 8824* (KUN0360171 & PE00987622–PE00987624); Chungtien, Sianrentang, 3500 m, [fl.], 18 July 1937, *T.T. Yü 12248* (KUN0360172 & PE00987648; PE00987885 [has been identified as *I. delavayi*])*; Chungtien, Wufengshan, grassland, 3000 m, [fl.], 31 July 1937, *T.T. Yü 12535* (KUN0360166 & PE00987625–PE00987627); Chung Tien [Plateau], Haba [Snow Mountain], 3500 m, [fr.], 8 September 1937, *T.T. Yü 13516* (PE00987632); Chungtien, Tungwahlung, margin of *Abies* forest, 3300 m, [fr.], 25 September 1937, *T.T. Yü 13590* (PE00987633 & PE00987646); Chungtien, Shianrentung, under forests, common, 3300 m, [fr.], 15 October 1937, *T.T. Yü 13711* (PE00987631 & PE00987899 [have been identified as *I. delavayi*]); Chungtien, Pica, mt. grassy slope, common, 3100 m, [fr.], 3 November 1937, *T.T. Yü 13935* (PE00987634–PE00987636); Upper Kiukiang Clolung Valley, Chialahmut, alpine swamp, 3800 m, [fr.], 7 August 1938, *T.T. Yü 19745* (KUN0360169 & PE00987611–PE00987613); Salwin Kiukiang Divide Panbahlung, side of mt. stream open and grassy place, 3000 m, [fr.], 22 October 1938, *T.T. Yü 20824* (KUN0360167 & PE00987605–PE00987607); Taron-Taru Divide, Barcuhwang, margin of alpine swamp, 2850 m, [fr.], 3 November 1938, *T.T. Yü 20943* (KUN0360138 & PE00987605–PE00987607); From Haba to Xuemenkeng in Shangri-La, open hillside, [fl.], 22 June 1939, *K.M. Feng 1352* (KUN0360177 & PE00987641), ibid. *K.M. Feng 1356* (KUN0360175 & PE00987642); Lijiang, Litiping between Likiang and Weihsi, on open margins of water, [fl.], 24 June 1939, *R.C. Ching 20889* (KUN0360174 & PE00987640); Shangri-La City, Haba Nichao, Haba Snow Mountain, Sanba Township, 3000–3500 m, [fr.], 8 October 1955, *G. Feng 21030* (KUN0360140

& PE00987898 [have been identified as *I. delavayi*])*; Lijiang City, Ludian Township Racecourse, wasteland grass, 3100 m, [fr.], 26 September 1956, *M. Pinyi 327* (PE00987890 [has been identified as *I. delavayi*])*; Yunnan, 2650 m, [fl.], 4 June 1957, *Z. Gao 1062* (PEM0009371 & PEM0009372)*; Yunnan, hillside, forest edge, grassland, 3200 m, [fl.], 10 June 1964, *S. Wu 7137* (KUN0360195)*; Small Zhongdian giant Pine forest, Gully, spruce, under the forest, 3240 m, [fl.], 12 June 1981, *Qinghai-Tibet Team 895* (CDBI0172816, CDBI0172817, HITBC050254, KUN0360146, PE01146641 & PE01146642)*; Shangri-La City, southeast of Mount Shika, 3730 m, [fl.], 3 July 1981, *Z. Tang et al. 529* (PE02238699)*; Shangri-La City, Dazhongdian, meadow pasture, 3300 m, [fl.], 18 July 1981, *Z. Tang et al. 645* (PE02238687 & PE02238688)*; Gongshan Dulong and Nu Autonomous County, to the Dulong River Bridge, hillside, moist, grass, ditch side, ~3000 м, [fl., fr.], 25 July 1982, *Qinghai-Tibet Team 8553* (KUN0360149, PE01146639, PE01146640 & PE01146646)*; Yunnan, subalpine meadow, 3200–3600 m, [fl.], 24 June 1989, *Z.D. Fang 1200* (SABG003760)*; 3 km south of Shangri-La City, plateau meadow, 3240 m, [fr.], 30 September 1992, *J. Chen 92406* (PE02238693)*; Zhongdian County, Beta Hai-Haba Shan junction, grassy meadows and marshes close to river, very common, 27º48'19"N 99º54'31"E, 3476 m, [fl.],18 June 1994, *ACE 404* (PE00987652); Yunnan, hillside, 4100 m, [fl.], 26 July 2000, *H. Wang s.n.* (KUN0360135)*; Shangri-La City, wet meadow, 27°53'55.2"N 99°43'54.78"E, 3300 m, [fr.], 7 October 2008, *Q.E. Yang 2716* (KUN1393127 & KUN1393128)*; Shangri-La City, coniferous and broad-leaved mixed forest, 27°26'22"N 99°48'33"E, 3322 m, [fr.], 24 August 2008, *Z.K. Zhou-07ZX-0154 et al.* (KUN1393107–KUN1393109)*; Deqin County, under fir forest, roadside, 27°37′21.6″N 99°41′21″E, 3450 m, [fr.], 16 September 2009, *Z. Jianwen et al. SunH-07ZX-2262* (KUN1393134 [has been identified as *I. clarkei*])*; Shangri-La City, roadside under the forest, 28°01′55″N 99°46′42.6″E, 3129 m, [fr.], 17 September 2009, *L. Guodong et al. SunH-07ZX-2285* (KUN1393133 [has been identified as *I. clarkei*])*; Shangri-La City, 27°38'35"N 99°38'22"E, 3849 m, [fr.], 7 October 2009, *Q.E. Yang 2858* (KUN1393102)*; Yulong Naxi Autonomous County, Xinzhu Village, on the way to the mountain, alpine meadow, 27°15'30"N 99°24'28"E, 3626 m, [fl.], 22 July 2009, *T. Zhang et al. 09CS1425* (KUN1393120–KUN1393122, as *I. bulleyana* f. *alba*); Shangri-La, Bigu Tianchi Lake, 3880 m, [fl.], 22 July 2009, *Y. Zhong 0103 & X. Quan* (PE02238683)*; Shangri-La City, wetlands, 27°48'18"N 99°48'40.3"E, 3410 m, [fr.], 15 September 2010, *J. Li 658 et al.* (KUN1393105)*; Shangri-La City, 27°53'52"N 99°43'34"E, 3322 m, [fr.], 2 October 2011, *X.K. Ou 11085 et al.* (KUN1393092)*; Deqin County, 28°18'52"N 99°07'30"E, 3773 m, [fr.], 6 October 2011, *X.K. Ou 11204 et al.* (KUN1393092)*. **Sichuan Province**: s. loc., [fl.], s.d., *S. Jiang et al. 10222* (PE00987590); [Kangding County], West Szechuen and Tibetan frontier, chiefly near Tachienlu, at 9000–13500 ft., [fl.], 17 June 1891, *A.E. Pratt 247* (P02163220 [has been identified as *I. delavayi*]); Bords des ruisseaux à La tsien lou, Lchéto, [fl.], July–August 1891, *R.P. Soulié 309* (P02163219 [has been identified as *I. delavayi*]); Thibet Oriental, Ta-tsien-lou (Principauté de Kiala), [fl.], 1893, *J. A. Soulié 517* (P02159139); [Left-hand side handwritten label]: Ta tsi lou, fl. bleu … formé, [fl.], 24 June [18]93, [Printed label]: Thibet Oriental, Ta-tsien-lou (Principauté de Kiala), 1893, *J. A. Soulié s.n.* Exs. No. 801 (K001273936! & P02159143, the original material of *I. delavayi*); Environs de Ta'tsienlou, sur les tombeaux, fleurs bleues, [fl.], 28 June [1893], [*J.A. Soulié*] *s.n.* (P02159136); Thibet Oriental, Tsékou (Haut Mékong), [fl.], 1893, *J. A. Soulié 1036* (P02159140 & P02159141); Tatsienlou, [fl.], 15 June 1898, *R.P. Missoy 360* (P02163226 & P02163227 [has been identified as *I. delavayi*]); Mountains between the Litang and Yalung rivers, between Muli Gomba and Baurong and Wa-Erh-Dje, 4300 m, [fl.], July 1928, *J.F. Rock 16632* (PE00033977 [has been identified as *I. delavayi*]); Mount Ta-Pao-Shan, between Wei-His and the Mekong, 3550 m,

[fl.], July 1928, *J.F. Rock 17132* (U1343205 [has been identified as *I. delavayi*]); Kangtin-ksien (Tatsienlu), in thickets, 9000–9500 ft. [3000–3166 m], [fr.], 25 September 1928, *W.P. Fang 3548* (PE00987872 [has been identified as *I. delavayi*]); Muli [Tibetan Autonomous County], Deongomba, mt. slope open place, very abundant, 3500 m, [fl.], 15 June 1937, *T.T. Yü 6260* (PE00987579–PE00987581); Muli, Wa-chin Nui-pen-tse, mt. slope grassy place, 3500 m, [fl.], 22 June 1937, *T.T. Yü 6569* (PE00987576–PE00987578); Jiulong County, Kiu-lung, Metikonga, Tsangpi, side of mt. stream, 3500 m, [fl.], 5 July 1937, *T.T. Yü 6851* (IBSC0629795 & PE00987568–PE00987570), ibid. *T.T. Yü 6858* (PE00987571 & PE00987572); Muli, Ku-lu, mt. slope grassy place, common, 3500 m, [fl.], 7 July 1937, *T.T. Yü 7145* (KUN0360159 & PE00987573–PE00987575); Muli, Kulu [Kangwu Dasi], side of mt. stream, grassy place, common, 3400 m, [fr.], 10 September 1937, *T.T. Yü 14228* (PE00987883 & PE00987884 [have been identified as *I. delavayi*]); Muli, Wachin, Sigenn, grassland, common, 3340 m, [fr.], 14 October 1937, *T.T. Yü 14541* (PE00987881 & PE00987882 [have been identified as *I. delavayi*]); Muli, Wachin, Jin-chang, grassland, common, 3500 m, [fr.], 17 October 1937, *T.T. Yü 14557* (PE00987584); Muli, Wachin, Jin-chang, mt. grassy slope, common, 3800 m, [fr.], 30 October 1937, *T.T. Yü 14676* (PE00987582 [has been identified as *I. delavayi*], PE00987587); [Sichuan], hillside, [fr.], 9 September 1939, *Z. Hechang 794* (NAS00555018 [has been identified as *I. delavayi*])*; Sichuan, [fr.], 1955, *Forestry Department 1* (PE00987566)*; Muli, 3800 m, [fr.], 03 October 1955, *Zhao 45* (PE00987564)*; Yanyuan County, 3100 m, [fl.], 11 June 1960, *S. Jiang 6003* (PE00987591)*; Jiulong County, foot of Wallong Mountain, 4000 m, [fl.], 3 June 1974, *Z. Liu 4726* (CDBI0169426, CDBI0169427 & CDBI0169475 [has been identified as *I. delavayi*])*; Muli, Bagua Commune, under the *Picea* forest 3100 m, [fr.], 25 August 1974, *Qinghai-Tibet Team 1876* (PE00987902 & PE00987903 [have been identified as *I. delavayi*])*; Sichuan, 4800 m, [fl.], 6 August 1978, *Q. Zhao 6472* (CDBI0169428, CDBI0169429 & CDBI0169680)*; Muli Tibetan Autonomous County, Yazui Forest Farm No. 2, hillside, under the *Abies* forest, 3400 m, [fr.], 19 August 1983, *Qinghai-Tibet Team 13005* (PE01146671 [has been identified as *I. delavayi*])*, ibid. *Qinghai-Tibet Team 13010* (PE01146672 & PE01146673 [have been identified as *I. delavayi*])*; Muli, Duck Tsui forest, hillside shrub grassland, 3800 m, [fr.], 22 August 1983, *Qinghai-Tibet Team 13241* (PE01146635 & PE01146636)*; [Zoigê County], 3500 m, [fl.], 10 July 1993, *Z. Tan 93-13* (PE02307996)*; Kangding, 29°56'00''N 101°58'17''E, 3318 m, [fr.], 26 August 2008, *X.F. Gao 0854* (KUN1393110–KUN1393113)*; Kangding, Zheduo Mountain, under sparse forest on both sides of the highway, 3500 m, [fr.], 13 September 2008, *Y. Zhong 0027 & H. Sun* (PE02238684)*; Kangding, Yulin Township, shady roadside, alpine grassland, 3300 m, [fr.], 27 September 2008, *Y. Zhong 0029 & H. Sun* (PE02238685)*; Xiangcheng County, woodside grass, 29°19'48.60''N 99°46'23.70''E, 3799 m, [fr.], 19 September 2009, *Y.P. Yang-Q-2213 et al.* (KUN1393087)*; Jiulong County, wetland, 29°16'20.7''N 101°28'18.6''E, 3860 m, [fr.], 29 September 2011, *D.C. Zhang-07ZX-2433 et al.* (KUN1393089)*; Mianning County, grass slope, 28°57'53.1''N 102°09'45''E, 2970 m, [fr.], 1 October 2011, *D.C. Zhang-07ZX-2465 et al.* (KUN1393104)*; Muli Tibetan Autonomous County, [fl.], 3 August 2011, *S. Yu et al. 4876* (PE02238689)*; Kangding, alpine grassland roadside sloping shrubs, 29°55'34.62''N 101°57'44.13''E, 3184 m, [fr.], 24 September 2012, *G. Yundong et al. GaoXF-12-176* (CDBI0253720 & KUN1393148 [have been identified as *I. delavayi*])*. **Xizang Province**: Nyingchi, Bayi District, Dongjiu, ditch side, 3700 м, 7 June 1966, *J. Zhang & J. Wang 0284* (PE01521044 & PE01521049)*; Nyingchi, Bayi, Tanabe, 3070 m, [fl.], 31 May 1972, *Tibet Chinese Herbal Medicine Survey Team 3137* (PE01521050)*; Mainling County, Xialonggou, hillside grassland, 3400–4600 m, [fl.], 17 July 1972, *Tibet Chinese Herbal Medicine Survey Team 4024*

(PE01521047)*; Nyingchi, east slope of Shejila, 4510 m, [fl.], 2 August 1975, *Qinghai-Tibet team 751074* (KUN0360206)*; Zayü County, ravine shrub grassland, 3700 m, [fr.], 8 September 1982, *Qinghai-Tibet Team 10165* (KUN0360205 & PE01146645)*; Nyingchi, Mainling, clearing between pine forests and shrubs, 29.11829°N 93.86555°E, 3050 m, [fr.], 13 September 2003, *X. Gao et al. 7406* (CDBI0190231 [has been identified as *I. delavayi*])*; Nyingchi, Bayi District, 29°48'14.4"N 94°44'49.9"E, 3174 m, [fr.], 18 September 2007, *Z. Ting et al. SCSB-B-000499* (KUN1393116–KUN1393118 [have been identified as *I. forrestii*])*; Nyingchi, Bayi District, 29°39'30"N 94°43'26"E, 3842 m, [fl.], 24 July 2008, *J. Luo 08XZ-084 & S. Wang* (HNWP00002828, KUN1393114 & KUN1393115)*; Nyingchi, Bayi District, alpine meadow, 29°48′28″N 94°44′34″E, 3080 m, [fr.], 26 September 2009, *S. Hang et al. SunH-07ZX-2719* (KUN1393135 [has been identified as *I. clarkei*])*; Nyingchi, Bayi District, meadow, 29°46'00"N 94°44'09.7"E, 3332 m, [fr.], 31 August 2011, *Y.P. Yang-Q-4056 et al.* (KUN1393103)*; Nyingchi City, Nyingchi County, Lunang Town, Mt. Serkyim La, slopes, 29°38'30.61"N 94°41'49.83"E, 3971 m, [fr.], 17 September 2017, *PE-Xizang Expedition PE6827* (PE01891560–PE01891562 [have been identified as *I. delavayi*]). **MYANMAR**. Upper Burma, N′Maikha [N′Mai River] – Salwin [Salween River] divide, [fl.], 1924, *G. Forrest 25043* (IBSC0628574); Upper Burma, N′Maikha – Salwin divide, [fl.], [June] 1925, *G. Forrest 26948* (IBSC0628575);

Iris chrysographes Dykes.—[Specimens from a cultivated plants], *Iris chrysographes* from W.R. Dykes, Bot. Mag., [fl.], 7 Jun 1911, *s.coll. s.n.* (K000802352!); Cult. Cambridge Bot. Garden, [fl.], 25 Jun 1932, W.T. Stearn s.n. (PE01679792). **CHINA**. **Yunnan Province**: N′Maikha – Salwin divide, [fl.], 1924, *G. Forrest 25043* (PE00987701); Marshy meadows and by mountain streams on the Chimi-li western flank of the N′Maikha Salwin divide, this may be only a form of *I. delavayi* but seems very distinct to the Yunnan type, 26°24'N 98°48'E, 13000 ft., [fl.], June 1925, *G. Forrest 26948* (PE00987702); W. Yunnan, [fr.], 1930, *G. Forrest 28762* (PE00987699); W. Yunnan, [fr.], 1930, *G. Forrest 29214* (PE00987697); [Yunnan], [fl.], s.d. [1931], *G. Forrest 30012* (PE00987698); Wei-si Hsien [Weixi Lisu Autonomous County], ravine side, 2300, [fl.], June 1935, *C.W. Wang 63721* (IBSC0628582 & NAS00555015); Wei-si Hsien, meadow, 3200 m, [fl.], August 1935, *C.W. Wang 67662* (IBSC0628584); Chungtien, Lichiashica, ditch side, 3300 m, [fl.], 17 June 1937, *T.T. Yü 11650* (IBSC0628585)*; Eryuan County, [Sanying District], Nandaping Village, meadow, 2900 m, [fr.], 31 July 1963, *Northwest Yunnan Jinsha River Team 6327* (KUN0360237, PE00987694 & PE00987695)*; North slope of Xuepan mountain [Lanping Bai and Pumi Autonomous County], subalpine meadow, 2800 m, [fl.], 17 June 1981, *Hengduan Mountain Team 590* (PE01479447, PE01479448, PE01146652 & PE01146653)*; Lanping County, 104 Forest Farm, subalpine meadow, 3100 m, [fl.], 28 June 1981, *Hengduan Mountain Team 802* (PE01479445, PE01479446, PE01146650 & PE01146651)*; Weixi Lisu Autonomous County, subalpine meadow and marsh meadow, dominant species, 3100–3700 m, [fl.], 19 July 1981, *Hengduan Mountain Team 1652* (PE01146647–PE01146649 & PE01479442–PE01479444)*; Lijiang City, Yulong Mountain [Jade Dragon Snow Mountain], wetland under the spruce forest, 3100–3300 m, [fl.], 4 August 1981, *Hengduan Mountain Team 2602* (PE01479449)*; Shangri-La City, eastern slope of Haba Snow Mountain, subalpine meadow in the fir forest, 3600–3700 m, [fl.], 11 August 1981, *Hengduan Mountain Team 2959* (PE01479450)*; [Yunnan], 3300 m, [fl.], 26 July 1982, *Z. Yang 82062* (IBSC0628581)*; [Yunnan], 4100 m, [fr.], 12 August 1986, *Z.N. Chen 16* (IBSC0628580)*; [Yunnan], hillside & open wet meadow, 3200–3310 m, [fl.], 23 June 2001, *s.coll. 615* (KUN0360234 & KUN0360236)*. **Sichuan Province**: Kangding County, [fl.], s.d., *s.coll. 790* (PE00987689); [Sichuan], [fr.], s.d., *Sichuan Economic Plant Expedition Team 21060* (CDBI0169448 & CDBI0169449; CDBI0169473 [has been

identified as *I. delavayi*])*; W. Sichuan, [fr.], August 1903, *E.H. Wilson 4555* (IBSC0628586); West of Kuan Hsien [Dujiangyan City], fl. violet, thickets, common, 7–11000 ft., [fl.], June [19]08, *E.H. Wilson 1304* (K000499091!, lectotype of *I. chrysographes*; BM000832587!, E00381804! & LE01011525!, isolectotypes); Mountains around Muli, [fl.], August 1930, *G. Forrest 28446* (PE00987700); Sichuan, [fl.], 1934, *G. Qu 829* (IBSC0628587)*; Kangting (Tachienlu), mont. occid., in prato subhumido, 3300 m, [fl.], 15 July 1934, *H. Smith 10578* (PE00987690); S. Mu-li, Wu-chin, Wa-ching-se, side of mt. stream, very abundant, 4000 m, [fl.], 20 June 1937, *T.T. Yü 6503* (KUN0360308 & PE00987878–PE00987880 [have been identified as *I. delavayi*]); Muli, Yangai, side of mt. stream & alpine swamp, 3300 m, [fl.], 29 June 1937, *T.T. Yü 6650* (KUN0360313 & PE00987875–PE00987877 [have been identified as *I. delavayi*]); Kang-ting hsien [Kangding], 3200 m, [fl.], 16 June 1953, *X. Jiang 36020* (IBSC0628583)*; Kangding County, 20 km highway in Zheduo Mountain, forest, 3700 m, [fr.], 17 August 1953, *X. Jiang 36624* (PE00987874 [has been identified as *I. delavayi*])*; Mianning County, Tuowu, waste grassland, 2400 m, [fr.], 16 July 1957, *S. Wu 2006* (PE00987688)*; Meigu County, hillside, [fr.], 23 August 1959, *J. Chuan 1997* (PE00987685)*; Yanyuan County, meadow at the bottom of valley, 3100 m, [fl.], 11 June 1960, *S. Jiang 5995* (PE00987682)*; Huangmaogeng, subalpine meadow, 3200 m, [fl.], 12 June 1960, *S. Jiang 7635* (PE00987681)*; [Daocheng County], 3950 m, [fl.], 1 June 1973, *Sichuan Vegetation Investigation Team 1832* (CDBI0169825 & CDBI0169476–CDBI0169478 [have been identified as *I. delavayi*])*; Daocheng County, Riwa Commune, grass ditch, 4450 m, [fl.], 25 June 1973, *Sichuan Vegetation Investigation Team 2233* (CDBI0169434, CDBI0169435, CDBI0169690, KUN0360240 & PE00987687)*; Jiulong County, foot of Wallong Mountain, 4000 m, [fl.], 3 June 1974, *Z. Liu 4726* (PE00987686)*; [Sichuan], 3600 m, [fl.], 2 July 1978, *Q. Zhao et al. 6851* (CDBI0169439, CDBI0169446 & CDBI0169688)*; [Sichuan], 3450 m, [fl.], 2 July 1978, *Y. Yang 7170* (CDBI0169438, CDBI0169442 & CDBI0170107)*; [Sichuan], 4700 m, [fr.], 31 July 1978, *Q. Zhao 6356* (CDBI0169432, CDBI0169447 & CDBI0169689)*; [Sichuan], 3870 m, [fl.], 2 August 1978, *Q. Zhao et al. 8058* (CDBI0169440, CDBI0169441 & CDBI0169687)*, ibid. 3800 m, [fr.], 2 September 1978, *Q. Zhao et al. 8078* (CDBI0169433, CDBI0169445 & CDBI0169685)*, ibid. 3500 m, [fr.], 8 September 1978, *Q. Zhao et al. 8467* (CDBI0169443, CDBI0169444 & CDBI0169686)*; [Sichuan], 3950 m, [fr.], 1 September 1979, *Q. Wang 29188* (CDBI0169451–CDBI0169453)*, ibid. 3500 m, [fr.], 8 September 1979, *Q. Wang 21131* (CDBI0169455, CDBI0169456 & CDBI0169474 [has been identified as *I. delavayi*])*; [Sichuan], 3050 m, [fr.], 8 October 1980, *Y. Cao & W. Zeng 33* (CDBI0172812 & CDBI0172813)*; [Sichuan], 3500 m, [fr.], October 1982, *Y. Yang et al. 210* (CDBI0172814 & CDBI0172815)*; [Sichuan], 1800 m, [fl., fr.], 11 June 1987, *Z. Zhao & J. Gao 87-0907* (WCSBG Nos. 009244–009249)*; Mao County, Fengyi Town, 2800 m, [fl.], 3 July 2007, *C. Zhang 20071130* (PE02238265, WCSBG Nos. 015437 & 015438)*; [Sichuan], [fr.], 2 July 2015, *W. Lin LW20150701* (CSH0106932)*. **Xizang Province**: Nyingchi, Bayi, Tanabe, 3070 m, [fl.], 31 May 1972, *Tibet Chinese Herbal Medicine Survey Team 3137* (HNWP Nos. 31482 & 82058, PE00987703)*; [Xizang], 3000 m, [fl.], 14 July 1972, *Tibet Chinese Herbal Medicine Survey Team 3933* (HNWP Nos. 32979 & 82190)*; Mainling County, Xialonggou, hillside grassland, 3400–4600 m, [fl.], 17 July 1972, *Tibet Chinese Herbal Medicine Survey Team 4024* (HNWP Nos. 33090 & 82330, PE00987704)*; [Xizang], 3000 m, [fl.], 3 June 1973, *Tibet expedition 287* (HNWP Nos. 37349 & 83492–83495)*; Nyingchi, east slope of Shejila, 4510 m, [fl., fr.], 2 August 1975, *Qinghai-Tibet team 751074* (HNWP Nos. 51692 & 96452, PE00987705–PE00987707)*; Mainling County, Doxiongla Mountain north slope, spruce, fir forest wetland and alpine pine forest waterside, 3200–3600 m [fl.], 20 June 1980, *s.coll. 10206* (PE01376829 & PE01376830)*;

Nyingchi, near Lulangbing Station, forest swamp meadow, 3100 m, [fr.], 16 September 1980, *Z. Ni et al. 1689* (PE00987712 & PE00987713)*; Mainling County, Deyang Commune, *Abies* forest edge meadow, 3600 m, [fl.], 19 July 1983, *B. Li & S. Cheng 5577* (PE02098420, PE00987710 & PE00987711)*; Nyingchi, one of the dominant species of meadow in forest, 3400–4000 m, [fl., fr.], 9 August 1983, *B. Li et al. 6400* (PE02098418 & PE00987708)*; Bayi District, roadside, 29°33'57.5''N 94°30'40.1''E, 3093 m, [fr.], 28 September 2009, *D.C. Zhang-07ZX-1847 et al.* (KUN1393129)*; [Xizang], 3600 m, [fl.], 16 July 2012, *C. Wang LZ098* (BNU0023177)*; [Xizang], roadside bushes, 4200 m, [fl.], 2 August 2016, *L. Wei & Y. He BNUXZ2016584* (BNU0028133)*.

Iris forrestii Dykes.—**CHINA**. **Yunnan Province**: Li Chung, half way up the mountain, [fl.], s.d., *McLaren 48* (P02158495); Yunnan, [fr.], s.d., *s.coll. s.n.* (PE01012191)*; Baoshan, Gaoligong Mountains, [fl.], s.d., *T.T. Yü s.n.* (PE01012209)*; Open mountain meadows on the eastern flank of the Lichiang [Lijiang] Range [Jade Dragon Snow Mountain], 27°30'N, 11–12000 ft., [fl.], June 1906, *Forrest 2426* (E00381810!, lectotype of *I. forrestii*; P02158497, isolectotype); Open alpine pasture on the eastern flank of the Lichiang Range, 27°20'N, 12–13000 ft., [fl.], June 1910, *G. Forrest 6028* (E00175209!, IBSC0628664, K!, P02158498 & PE01012551); Prope urbem Lidjiang, imprimis in monte Yülung-schan, [fl.], [1914–1915], *Handel-Mazzetti 4076* (P02158496); Between Likiang, Youngning, and Youngpei, [fl,], June 1922, *J.F. Rock 5219* (KUN0360336); Mount Habashan, north of Ndaku, north of the Likiang Range, Yangtze drainage basin, 12000 ft., [fl.], July 1923, *J.F. Rock 9652* (KUN0360335); Heqing County, Zhaobi Mountain, Lianping, 2700, [fr.], 24 August 1929, *R. Qin 24043* (PE01012196)*; W. Yunnan, [fl.], 1930, *G. Forrest 28655* (PE01012549), ibid. [fr.], *G. Forrest 29154* (PE01012550); [Shangri-La City], alpine meadows north of Chung-tien in Tonwa Territory, 11500 ft. [~3000 m], [fl.], May 1932, *J.F. Rock 24708* (IBSC0628663 & NAS00555112); Lan-ping Hsien [Lanping Bai and Pumi Autonomous County], by stream, 3600 m, [fr.], 17 August 1933, *H.T. Tsai 53992* (IBSC0629113 [has been identified as *I. wilsonii*] & PE00987894 [has been identified as *I. delavayi*]); Wei-si Hsien, [fl.], June 1935, *C.W. Wang 63721* (IBSC0629811)*; Lijiang City, Xuesong Village, grass slope, 3200 m, [fl.], 22 Juyl 1937, *T.T. Yü 15342* (PE01012203–PE01012205)*; Chungtien Plateau, 3000 m, [fr.], 5 October 1937, *T.T. Yü 13617* (PE01012197–PE01012199); Deqin County, hillside meadow, 3400 m, [fr.], 28 October 1937, *T.T. Yü 10607* (PE01012206 & PE01012207)*; N. flank of Haba Snow Range, stream [fl.], 22 June 1939, *K.M. Feng 1353* (KUN0360332 & PE01012194); Likiang Snow Range, open pasture, [fl.], 5 July 1939, *R.C. Ching 30355* (KUN0360339 & PE01012211)*; [Gongshan Derung and Nu Autonomous County], Changpu, 3000–3400 m, [fr.], 9 September 1940, *K.M. Feng 7614* (PE01012195)*; Lijiang City, [Jade Dragon] Snow Mountain, [fl.], 17 July 1940, *R. Qin 30870* (KUN0360338 & PE01012193)*; Lijiang City, Yulong Naxi Autonomous County, swamp meadow, 3200 m [fl.], 6 June 1985, *Sino-British Expedition Team 85-595* (KUN0360328)*; Lijiang, Yulong Naxi Autonomous County, roadside, wet grass, hillsides and ditch, 3040–3150 m, [fl.], 26 June 2001, *Y. Shen 634* (KUN0360341)*; Lijiang, Yulong Naxi Autonomous County, moist alpine meadow, 2950 m, [fl.], 3 June 2001, *Y. Shen 10539* (KUN0360340)*; Lijiang, Yulong Naxi Autonomous County, Gelangda, 27°03'41.2''N 99°46'36.5''E, 3649 m, 9 Aug 2012, *Yulong County Census Team 5307210305* (IMDY0024336)*. **Sichuan Province**: Mountains around Muli, [fl.], June 1930, *G. Forrest 28420* (PE01013880 [has been identified as *I. wilsonii*]); Muli, [fl.], [1930], *G. Forrest 30626* (PE01012210); Hsi-chang Hsien, mt. slope moist place, fl. yellow with purple lines, 2900–3100 m, [fl.], 10 August 1932, *T.T. Yü 1298* (PE01012190); S. Kiulung [Jiulong County], Metikonga, Tsangpi, side of mt. stream, 4100 m, [fl.], 5 July 1937, *T.T. Yü*

6849 (KUN0360330; PE00034105, PE01013871 & PE01013872 [have been identified as *I. wilsonii*]); Muli, Dao-lang-kang-din, margin of woods, rare, 2800 m, [fl.], 1 August 1937, *T.T. Yü 7528* (IBSC0628662, KUN0360329 & PE01012200–PE01012202); Muli County, Wachin, near Lama, mt. grassy slope, 3100 m, common, [fr.], 7 October 1937, *T.T. Yü 14435* (PE01013873 [has been identified as *I. wilsonii*]); Muli, Lilang, mt. grassy slope, 2600 m, common, [fr.], 19 December 1937, *T.T. Yü 14883* (PE01012186–PE01012188); Sichuan, grass slope forest edge, 3200 m, 26 July 1958, *S. Chen et al. 10911* (NAS00555113)*; Yanbian County, China Rock Township nine bamboo forest, hillside forest, 2700 m, [fl.], 25 June 1983, *Qinghai-Tibet Team 11470* (PE01146706 & PE01146707 [have been identified as *I. wilsonii*])*; Miyi County, White Hill, pine and alpine oak forest margin, 3000–3300 m [fl.], 7 July 1983, *Qinghai-Tibet Team 11801* (PE01146679 & PE01146680)*; Yanyuan County, Yuanbao District, Huolu Mountain, hillside shrub grassland, 3800 m, [fl.], 21 July 1983, *Qinghai-Tibet Team 12235* (PE01146710 [has been identified as *I. wilsonii*])*, ibid. 24 Jul 1983, *Qinghai-Tibet Team 12417* (PE01146711 [has been identified as *I. wilsonii*])*.

Iris wilsonii C.H.Wright.—**CHINA**. [Specimens from cultivated plants], China [Hubei Province, Fang County], Wilson, Kew, [fl.], 26 June [19]07, *s.coll. 1164a* (K000499094!, lectotype of *I. wilsonii*), ibid. *s.coll. 1164* (K000499095!, isolectotype); Rootstock and leaves of Chinese iris sent to Kew for determination, June 1907, *s.coll. s. n.* (K000499096!, the original material of *I. wilsonii*); [Chongqing, Wushan County, 6500–10000 ft.], Kew Bulletin, 1907, p. 321, [fl.], *s.coll. 1450* (K000499093!, the original material of *I. wilsonii*); [China], 1900 m, [fr.], 24 September 1957, *Q. Hu 663* (LBG00106663 & LBG00106664)*. **Hubei Province**: Fang Hsien [Fang County], 7000 ft., [fl.], 8 April [1909], fl. yellowish, *E.H. Wilson 3072* (K001273937!, epitype of *I. wilsonii*); Badong County, [fr.], 22 September 1958, *M. Qian 1050* (PE01013868)*; Shennongjia, hillside, 2400 m, [fr.], 23 June 1976, *Shennongjia Plant Examination Team 10619* (PE01056853 & PE01056854)*; Shennongjia, hillside, 2800 m, [fl.], 9 July 1976, *Shennongjia Plant Examination Team 10731* (PE01056855 & PE01056856)*; Shennongjia, Xiaoqianjiaping, 1850–1900 m, [fl.], 20 July 1976, *Shennongjia Plant Examination Team 31168* (PE01056852)*; Shennongjia, vicinity of Xiao Shennongjia, 31°30'N 110°30'E, 2700–2900 m [fr.], 10 September 1980, *Sino-American Botanical Expedition 952* (KUN0360641, NAS00555577 & PE01056877); Shennongjia, wetland meadow among grass, 31°43'N 110°26'E, 2000 m [fr.], 15 Novemebr 2008, *J.P. Li 5* (KUN1393311 & KUN1393312)*; Shennongjia, hillside grass, forest edge grassland and wetland beside river and ditch, [fl.], 19 June 2012, *D. Zhang 12061909* (JIU23141)*. **Sichuan Province**: [Sichuan], [fl.], s.d., *T.H. Tu 829* (IBSC0629114); [Leshan], 9000 ft. [~2700 m], [fl., fr.], July 1904, *E.H. Wilson 4556* (BM000832589! & IBSC0629112); Henshe?, 11000 ft., [fl.], [1904?], *E.H. Wilson 4557* (IBSC0629111); Juei-she Hsien [Yuexi County], 2200 m, [fr.], 3 June 1932, *T.T. Yü 965* (CQNM0006910 & PE00987691 [has been identified as *I. forrestii*]); Ebian Yi Autonomous County, [fr.], 26 August 1939, *S.L. Sun 1084* (PE01013879)*; Meigu County, swampy marsh, 2400 m, [fl.], 12 July 1959, *J. Chuan 1066* (PE01013876 & PE01013877)*; Leibo County, grass, 3100 m, [fl.], 20 June 1959, *J. Chuan 848* (KUN0360343 & PE01012183 [have been identified as *I. forrestii*])*; Leibo County, 288 km away from Huangmaogeng, upper alpine meadow hillside, 3000–3300 m, [fl.], 20 June 1959, *Z. Guan 8618* (PE01012184 & PE01012185 [have been identified as *I. forrestii*])*; Zhaojue County, hillside slope, 2170 m, [fl.], 26 June 1960, *W. Sun 15* (SZ00042889–SZ00042895 [have been identified as *I. forrestii*])*; Zhaojue County, Lanba Township, wet ditch, 2500 m, [fl.], 30 June 1976, *Sichuan Vegetation Team 12818* (CDBI0169479, CDBI0169480 & CDBI0169824 [have been identified as *I. forrestii*]; CDBI0170168 & PE01146709)*; Mianning County, Tuowu Commune, river valley,

2100 m, [fl.], 10 June 1976, *Xishi Biology 11765* (CDBI0169676, CDBI0169677, CDBI0170167 & PE01146708)*; Liangshan, Meigu County, near marker post 322 S of Yizhui Pass on Daliang Shan, between Maigu and Ebian, on gravelly loam in a flat wet open grazed meadow, ca. 2290 m, [fl.], 18 October 1992, *Sichuan Expedition 1229* (E00212134!, cult. No. 15035). **Yunnan Province**: [Shangri-La], open moist stony pastures on the Chungtien plateau, 27°55'N, 10000 ft., 3300 m, [fl.], July 1913, *G. Forrest 10675* (PE01013882); Open dry rocky hillsides and meadows on the Chienchuan–Mekong divide, 26°20'N 99°20'E, 11000 ft., [fl.], July 1923, *G. Forrest 23611* (PE01013881); Weisi Lisu Autonomous County, meadows, 3500–3600 m, [fl.], 19 July 1981, *Hengduan Mountain Team 1680* (PE01146704, PE01146705, PE01479455 & PE01479456)*. **Shaanxi Province**: Yang County, [fl.], 7 August 1938, *T.N. Liou & P.C. Tsoong 2998* (PE01013867); Fuping County, Huangcaoping, bank near the wet place, [fl.], 7 July 1952, *B. Guo 1597* (CDBI0169674 & PE01013866)*; [Shaanxi], 1700 m, [fl.], 14 June 1977, *K. Fu 17528* (IBK00142763 & PE02238520); Mount Taibai, hilltop meadow, 2086 m, [fl.], 10 July 2011, *S. Li et al. 15622* (XBGH006770)*; Ningshan County, subalpine wetland grass, 33°28'20.84"N 108°29'48.81"E, 2106 m [fl.], 12 July 2014, *X. Tian 1117 & M. Tian* (KUN1393310)*. **Gansu Province**: Hui County, roadside near the forest, 2100 m, [fr.], 17 July 1950, *Z. Zhang 839* (KUN0360642)*. **Chongqing municipality**: Wuxi County, Lanying Village, Shuanghe Township, grass slope, 2700 m, [fl.], 17 July 1958, *G. Yang 58852* (IBK00283003, PE01013875 & PE02238575)*; Chengkou, 1950 m, [fl.], 27 June 1958, *T. Dai 101051* (IBK00381808 & PE01013870)*; Chengkou, 2000 m, [fr.], 19 October 1958, *T. Dai 106754* (PE01013874 & PE02238297)*; Golden Buddha Mountain [Nanchuan District, Mount Jinfo], in the grass by the mountain pond, 2050 m, [fl.], 19 July 1977, *Z. Liu 771338* (IMC0013985)*.
Iris delavayi Micheli.—[Specimen from a cultivated plant], Haarlem, van Tubergen, [fl.], 24 June 1949, *B.K. Boom s.n.* (L1472280). **CHINA**. s.loc., [fl.], s.d., *G. Forrest 29992* (PE00987901). **Yunnan Province**: Fl. bleues, lieux humide[s], sur les versants du Lo pin chan (Lan Kong) à 2800 m d'altit., [fl.], 4 July 1886, *J.M. Delavay 2136* (P02163221, P02163223 & P02163230); Fl. bleues, lieux marécageux du Hia lopin (Lan Kong) à 2800 m d'altit[ude]., [fl.], 4 July 1886, *J.M. Delavay 2365* (P02163222); Fl. violetts, marais de Kan-hay-tze, sur le Hee chan-men à 2800 m. d'altit., [fl.], 14 June 1887, *J.M. Delavay 2683* (K!, L1472279, P02163231–P02163233); Fl. bleue, marais, sur le Yu Kia Ngan, au dessus de Pon Man tsen, á 2800 m, [fl.], 3 July 1888, *J.M. Delavay 4808* (P02159142 & P02159146 [have been identified as *I. bulleyana*]); Fl. bleues, marais, sur le Tsang Chan à 2500 m d'alt., [fl.], 15 June 1889, *J.M. Delavay 4164* (P02163234 & P02163235); Dali City, near Wuwei Temple, forest by the water ditch, [fl.], 21 May 1929, *R. Qin 22710* (KUN0360310 & PE00033976)*; [Dali], wet grass slope, [fr.], 18 July 1929, *R. Qin 25066* (KUN0360309)*; Ta-li Hsien [Dali City], meadow, spring side, 2800 m, [fl.], May 1935, *C.W. Wang 63151* (PE00987891 & PE00987892); Ta-li Hsien, flat slope, 2540 m, [fl.], May 1935, *C.W. Wang 63481* (PE00987696 [has been identified as *chrysographes*]); [Yunnan], [fl.], July 1941, *H. Wang 1029* (PE00987896); Dali City, near Cangshan Zhonghe Temple, [fr.], 30 November 1941, *S. Liu 19290* (PE00987887)*; Yangbi Yi Autonomous County, Maidi East, [fl.], 19 June 1942, *H. Wang 23921* (PE00987889)*; Dali City, mid-levels of Malong Peak, [fl.], 3 July 1943, *H. Wang 3949* (PE00987888)*; Dali City, Cangshan Zhonghe Temple, [fr.], 2 October 1946, *S. Liu 20934* (PE00987886)*; Dali City, eastern slope of Cangshan Mountain, 2300–2650 m, [fl.], 30 May 1955, *Z. Wu et al. 1396* (PE00987897); Dali City, Cang Mountain, by the side of a ditch, grassland, 2800 m, [fr.], 3 August 1963, *s. coll. 63-3831* (KUN0360311)*; [Yunnan], 2950 m, [fl.], 31 May 1982, *B. He 82083* (LBG00106679–LBG00106681)*; Dali Xian, E side of Diancang Shan mountain range, vicinity of Yinglofen, 2900–3050 m, flowers purple, [fl.], 10

July 1984, *Sino-American Botanical Expedition 959* (KUN0360312, epitype of *I. delavayi*); Dali Bai Aut. Pref., Dali Co., Tsang Shan, Little Huadianba, in a stream, 3100 m, [fl.], 19 October 1990, [*E.J. Cowley*] *1561* (E00328214!, cult. No. 15833). **Xizang Province**: Hawu to Yadong, by the stream, [fl.], 23 July 1953, *B. Zhong 5919* (PE00987904 & PE00987905)*; Yadong County, from Lao Yadong to Xia Yadong, meadow, 3000 m, [fl.], 23 May 1961, *J. Zhang 2480* (PE01521046 [has been identified as *I. bulleyana*] & PE00987714 [has been identified as *chrysographes*])*; Yadong County, Asan Village, 2900 m, [fl.], 31 May 1975, *Qinghai-Tibet Team 750083* (HNWP Nos. 50717 & 96925 [have been identified as *I. chrysographes*]; KUN0360207, PE00987655 & PE00987656 [have been identified as *I. bulleyana*]); Yadong County, Zuokubu Mountain Pass, waterside glade, 3300 m, [fl.], 12 June 1975, *Qinghai-Tibet Team 750300* (PE00987906 & PE00987907)*; Yadong County, 3400 m, [fl.], 24 June 1975, *s.coll. 7086* (PE01521045 [has been identified as *I. bulleyana*])*; Yadong County, Xiayadong Township, 27.408792N 88.970308E, 2808 m, [fl.], 17 June 2018, *PE–Xizang Expedition Team 7448* (PE02332323 & PE2332479)*.

Iris clarkei Baker ex Hook.f.—**INDIA**. **Sikkim State**: Sikkim Himal., [fl., fr.], [1848], [*Hooker*] *s.n.* (K000098495!, lectotype of *I. clarkei*; Sikkim, [fl., fr.], [1848], [*Hooker*] *s.n.* (K001273940! & K001273941!, original material); *Iris* ??, s. loc., 15 May [18]48, [*Hooker*] *s.n.* (K001273939!, the original material of *I. clarkei*); Sikkim, [fr.], 14 October 1868, *S. Kurz s.n.* (P02159922); Sikkim, Jakla, 11500 [ft.], [fr.], 17 October 1869, *C.B. Clarke 10100* (K001273935!, original material); Sikkim, [fl.], 1877, *G. King 4467* (L1472263 & P02159920); Chola Range, Natang [Nathang Valley], 12000 ft., [fr.], 30 September 1892, *G.A. Gammie s.n.* (L1472262 & P02159923); Changu [Tsomgo Lake], 12300 ft. [~3750 m], [fl.], 13 July 1956, *D. Chatterjee 196* (AMD75608). West Bengal State: Tonglo [Tonglu], Darjeeling, 10000 [ft.], 13 September 1875, *C.B. Clarke 27404* (K000098496!, the original material of *I. clarkei*). **NEPAL**: E. Nepal, Kangchejunga Mnts. (near Ghunsa), 2300 m, peduncle with a narrow cave, not totally solid but nearly so, falls with white, not yellow at all, [fl.], s.d., *s. coll.*, *s.n.* (L3912484, cult.); Open pastures above Trogsindho Pass, 3288 m, [fl.], 19 November 1984, *E.F. Needham 674* (E00037818!, cult.); Sagarmatha Zone, Solukhumbu District, Thuli Kharka, on damp meadows, 27°37′N 86°46′E, 3870 m, [fl.], 28 August 1997, *M. Wakabayashi et al. 97 30377* (KUN1261284 & L4367633). **CHINA**. **Yunnan Province**: Gongshan County, near Heiwadi, 27°45′40.5″N 98°36′05.7″E, 2429 m, [fr.], 27 August 2009, *F. Zhendong LDHF-579* (SABG000755)*. **Xizang Province**: Yadong County, hillside, 3150 m, [fr.], 23 July 1960, *F. Guoxun 935* (PE00987717)*; Lulangbing Station, grass waterside, 3020 m, [fr.], 27 July 1965, *Z. Yongtian & Y. Kaiyong 965* (PE00987716)*; Yadong County, near Chuntangtang, wet place in hillside shrub, 3980 m, [fl.], 14 September 1974, *Qinghai-Tibet Team 2506* (PE00987718 & PE00987719)*. **BHUTAN**. Thimphu [District], Wangdi Phodrang, Dochu La, in open ground or in mixed woodland, 3130 m, [fl.], 18 June 1990, *K. Rushforth 926* (E00607321!).
