# Supplementary material for: Taxonomic Composition of Iris Subser. Chrysographes (Iridaceae) Inferred from Chloroplast DNA and Morphological Analyses
Source: Plants (Basel). 2021 Oct 20;10(11):2232. doi: 10.3390/plants10112232 (PMC8621552; doi:10.3390/plants10112232)
Supplement: Supplementary file 1 [file plants-10-02232-s001.zip › Table S2.pdf]

Table S2. The results of the variance analysis of the *Iris* subser. *Chrysographes* species.

Character	PC1	PC2	PC3	*p*-value	Species	M±S	CV
LL	0.277	**0.431**	–0.002	0.002	*I. bulleyana*	30.4±12.4	41.0
				(0.0032)	*I. forrestii*	27.7±12.7	45.9
					I. chrysographes	33.2±11.9	35.9
					I. delavayi	46.0±11.8	25.6
LW	**0.389**	–0.131	0.096	<0.0001	*I. bulleyana*	0.5±0.2	36.0
				(<0.0001)	*I. forrestii*	0.3±0.1	38.2
					I. chrysographes	0.7±0.3	42.4
					I. delavayi	1.1±0.2	21.6
SH	**0.370**	–0.106	0.208	<0.0001	*I. bulleyana*	31.1±18.1	58.3
				(<0.0001)	*I. forrestii*	19.7±8.7	44.5
					I. chrysographes	34.9±12.9	36.8
					I. delavayi	71.4±28.2	39.5
CL	0.146	0.202	**–0.831**	0.102	*I. bulleyana*	9.4±1.9	20.3
				(0.102)	*I. forrestii*	11.2±5.1	45.9
					I. chrysographes	12.4±3.0	24.0
					I. delavayi	12.5±3.3	26.7
BL	0.328	0.247	**0.338**	0.0004	*I. bulleyana*	6.3±1.1	18.0
				(0.001)	*I. forrestii*	6.3±1.0	15.7
					I. chrysographes	7.0±1.3	18.0
					I. delavayi	8.0±0.8	10.1
PL	0.319	**0.591**	0.134	0.0014	*I. bulleyana*	3.3±1.3	38.1
				(0.0028)	*I. forrestii*	3.5±1.5	44.3
					I. chrysographes	4.4±1.5	33.1
					I. delavayi	5.8±2.4	40.2
						**Mode**	**var**
IS	**0.349**	**–0.492**	–0.199	–	*I. bulleyana*	–	–
					I. forrestii	–	–
					I. chrysographes	–	–
					I. delavayi	–	–
NF	**0.418**	–0.282	–0.160	0.0155	*I. bulleyana*	1	1–2
				(0.0206)	*I. forrestii*	1	1–2
					I. chrysographes	1	1–2
					I. delavayi	3	2–3
NC	0.328	–0.115	0.138	0.0239	*I. bulleyana*	1	1–2
				(0.0273)	*I. forrestii*	1	1–3
					I. chrysographes	1	1–2
					I. delavayi	2	1–5

M – mean (cm), S – standard deviation, CV – coefficient of variation (%), var – variation (minimum–maximum). The values in parentheses are adjusted *p*-values. A dash (–) indicates no analysis. Names of characters and their descriptions are provided in Table 2. Numbers in bold indicate absolute values of the factor loading above 0.33 in the first and in the second axes that contribute significantly to each principal component.
