# Supplementary material for: Taxonomic Composition of Iris Subser. Chrysographes (Iridaceae) Inferred from Chloroplast DNA and Morphological Analyses
Source: Plants (Basel). 2021 Oct 20;10(11):2232. doi: 10.3390/plants10112232 (PMC8621552; doi:10.3390/plants10112232)
Supplement: Supplementary file 1 [file plants-10-02232-s001.zip › Table S3.pdf]

Table S3. Nucleotide divergence between groups identified by the MJ (four haplogroups) and phylogenetic analyses (three clusters) of *Iris* subser. *Chrysographes* from 25 localities, and also between *I. bulleyana*, *I. forrestii*, and *I. chrysographes* (haplogroup A) and *I. delavayi* (haplogroup B) as inferred from the cpDNA data.

Group	Cluster I	Cluster II	Cluster III	Haplogroup A	Haplogroup B
Cluster I		16.263 (11)	15.263 (9)		
Cluster II	0.00435		16.500 (12)	16.714 (14)	15.000 (13)
Cluster III	0.00408	0.00440		15.714 (12)	14.000 (11)
Haplogroup A		0.00447	0.00419		5.714 (5)
Haplogroup B		0.00400	0.00374	0.00153	

Species	*I. bulleyana*	*I. forrestii*	*I. chrysographes*	*I. delavayi*
I. bulleyana		2.375 (0)	0.875 (0)	5.875 (5)
I. forrestii	0.00063		1.500 (0)	6.500 (6)
I. chrysographes	0.00023	0.00040		5000 (5)
I. delavayi	0.00157	0.00173	0.00133	

Above diagonal, average number of nucleotide differences (number of fixed differences); below diagonal, average number of nucleotide substitution per site (K_S).
